# A systematic review and content analysis of serious video games for children with ADHD

**DOI:** 10.3389/fpsyt.2025.1605744

**Published:** 2025-10-06

**Authors:** Brandon K. Schultz, Steven W. Evans, Kaitlynn M. Carter, Allison Dembowski, Kelly Lojinger, Emma R. Murray, Christy Walcott

**Affiliations:** ^1^ Department of Psychology, East Carolina University, Greenville, NC, United States; ^2^ Department of Psychology, Ohio University, Athens, OH, United States

**Keywords:** ADHD, digital health, serious games, systematic review, content analysis

## Abstract

Content analysis is a critical step in understanding any mental health treatment, but these details are absent in the serious video games for ADHD literature. To better understand specific ADHD game elements, we conducted a systematic review and identified 37 seminal studies, published between February 2005 and March 2021, investigating 22 distinct ADHD games (final search on January 16, 2025) designed for children and adolescents. We coded those studies and supplementary game descriptions for therapeutic game content, then calculated effect sizes for immediate post-treatment effects on parent ratings of ADHD symptoms (i.e., far transfer), where available. There appeared to be considerable content variability across titles, but most games in this review (55%) attempt cognitive training, with pluralities deploying the go/no-go, continuous performance, and Corsi block tapping task paradigms. Nearly one-fifth (18%) of the games include theta/beta ratio neurofeedback, and more than one-quarter (27%) of the games include novel content (e.g., physical exercise, eye gaze training). Changes in parent ratings of ADHD symptoms range widely (*d*s = -0.55 to 1.26) without an obvious pattern of advantage for any game element. The largest far transfer effects for ADHD games are found in study results at highest risk of bias, seemingly irrespective of game content. Our findings suggest that far transfer effects are unconvincing for seminal game elements, and that new directions in ADHD game design and delivery are warranted.

## 1 Introduction

Serious games are designed to alter player knowledge, abilities, or behavior, rather than to simply entertain (1). In recent decades, serious video games have been designed to evaluate and treat mental illnesses, with emerging research showing some clinical benefits (e.g., 2). The most consistent finding is that serious games appeal to consumers (3) and are safe, with one product recently receiving safety clearance by the U.S. Food and Drug Administration (4). But the extent to which digital products use proven therapeutic elements is unclear. Like other treatments, the research on serious games warrants careful review and analyses before assuming any are appropriate for clinical care. To this end, we conducted a systematic review and content analysis of serious games for a high-incidence disorder to assess the clinical benefits of specific game elements.

### 1.1 Gamified ADHD treatments

A subset of mental health games is targeted to the symptoms, impairments, or deficits of children and adolescents with attention-deficit/hyperactivity disorder (ADHD). ADHD is a neurodevelopmental disorder characterized by persistent patterns of inattention and/or hyperactivity-impulsivity that interfere with academic, vocational, or social functioning. Serious video games for youth with ADHD (hereafter, *ADHD games*) might be designed to provide established assessment and psychosocial treatment (see 5), or ADHD games might deploy experimental elements, giving rise to new interventions. In either case, an ADHD game and its attributes can be contrasted with professional guidelines to determine its alignment with current best practices. Powell and colleagues (6) conducted such an analysis with popular mobile apps for ADHD by inviting children and clinicians to review app content. Interestingly, clinicians raised concerns about the lack of research support for elements within the apps and expressed disappointment that the apps were not targeted to practical needs. But this examination was limited to popular commercial products, which are not necessarily informed by scholarly research.

To assess the degree to which research-based ADHD games build on professional guidelines, it is important to contrast game content with evidence-based practices (EBPs). Treatment guidelines for ADHD have been produced by the American Academy of Pediatrics (AAP; 7), the Society for Developmental and Behavioral Pediatrics (SDBP; 8), and the Society of Clinical Child and Adolescent Psychology (SCCAP; 9). Among non-pharmacological treatments, behavioral approaches—including parent training, classroom management, and peer interventions—are strongly supported (i.e., “well-established”). Skill training interventions, like organization training, are also well established, provided those strategies target pertinent skills, provide practice over time, and include performance feedback. At lower levels of support, the professional guidelines diverge; the SCCAP identifies neurofeedback (and biofeedback more broadly) as possibly efficacious and cognitive training as experimental, whereas the AAP and SDBP withhold recommendation. In short, behavior therapy (BT) and training interventions are strongly supported, whereas neurofeedback (NF) and cognitive training (CT) may hold promise but are not as substantiated.

It appears most ADHD games in the empirical literature deliver CT and/or NF (10, 11) which warrants special consideration given the limitations in the literature. ADHD is associated with neurocognitive deficits in reaction time, sustained attention/vigilance, working memory, and response inhibition, often assessed using computerized laboratory tasks (12). Discoveries in neuroplasticity imply these deficits are corrigible (13), and that improvements in these domains can alleviate ADHD symptoms and impairments. To that end, CT and NF have been designed to engage and strengthen specific neurocognitive functions. In the case of CT, treatments typically use laboratory assessment tasks repurposed as training exercises. The challenge is that training activities that are identical to the original laboratory tasks might lead to skill improvement (e.g., digit span performance) but not neurorehabilitation in a broader sense (e.g., improved working memory). Thus, CT developers often create training experiences using modified laboratory tasks that theoretically increase the transfer of training, but the degree to which a validated laboratory task can or should be modified for training purposes is unknown (14).

In recent decades researchers have attempted to gamify both CT and NF tools to increase user engagement and motivation. The resulting tools mostly improve user performance on laboratory measures, but rarely produce real-world outcomes observable by parents or teachers (15, 16)—a distinction referred to as *near* versus *far* transfer. In short, behavior changes in domains that closely resemble a training task are considered “near” transfer, and behavior changes in domains unlike a training task are considered “far” transfer (17). The failure for ADHD games to consistently achieve far transfer beyond game-like situations is still not understood. One potential explanation is that, despite an association with ADHD, neurocognitive deficits may be irrelevant to clinical outcomes (i.e., correlation ≠ causation) (18). Alternatively, because neurocognitive dysfunction varies considerably between individuals (19), each tool may only benefit specific ADHD subpopulations. Or current tools may not effectively target the core processes impaired in ADHD. Games billed as “cognitive training” or “brain training” for ADHD could be comprised of activities targeting secondary or even unrelated processes (20). In any event, it is critical that treatments deliver meaningful, real-world behavior change at home or school, and CT/NF efforts generally appear to fall short.

### 1.2 ADHD game elements

By operationalizing practices like CT, NF, and BT, it is possible to categorize ADHD game content. Clearly not all BT lend themselves to easy gamification (e.g., parent training), but training interventions (5, p. 730), like time management and organization skills coaching for children, seem particularly well-suited to gameplay. Training interventions target the functional impairments associated with ADHD and can be readily operationalized based on the skills taught (e.g., organization training). NF is also relatively straightforward to operationalize given its distinguishing targets and instrumentation (e.g., electroencephalography) (21). But the cognitive abilities targeted by CT, like working memory and response inhibition, are subjectively categorized. Moreover, it is difficult, if not impossible, to examine or remediate a cognitive process in isolation (12). So, rather than identifying the latent processes that CT games putatively target, we believe it is most useful to identify the laboratory task paradigms deployed in each. For example, “visuospatial memory” is ambiguously defined, but the Corsi block tapping task, often used to measure it, is readily identifiable. Game elements approximating these tasks are informed by the research literature, whereas unique and innovative content can be judged novel (i.e., untested prior to gamification).

ADHD games have been reviewed many times, but there are two major limitations with these previous efforts. First, most ADHD games do not advance beyond early development stages (e.g., proof-of-concept), and yet reviewers rarely distinguish between nascent efforts and influential games that garner widespread scholarly attention. And second, only one study to date has attempted to examine game content (6), and that effort was limited to popular mobile apps. A content analysis is consistent with the experimental therapeutics approach to treatment development promoted by the National Institute of Mental Health because it provides clarity regarding both target mechanisms (e.g., working memory) and their associated clinical effects (e.g., symptom reduction) (22). Content analysis is a critical step in understanding any mental health treatment (23), but this information is conspicuously lacking in the ADHD games literature.

### 1.3 The present study

We pursued three primary aims (1): identify seminal ADHD games repeatedly cited in published, systematic reviews (2); categorize the therapeutic content within these games; and (3) determine the game elements associated with the most promising effects on parent ratings of ADHD symptoms (i.e., far transfer). Many of the games reviewed are proprietary or limited to use outside the United States, so we were unable to play most ourselves. Instead, we relied on a systematic review to identify the games, then synthesized the data from repeatedly cited studies, and corroborated our results with the study authors. We discuss what our results suggest about the ADHD games literature and then offer our recommendations for advancing this research, particularly as it applies to school-based intervention efforts.

## 2 Method

The current project was supported in part by a grant to East Carolina University and Ohio University (R324A180219), and we adhered to the most recent Preferred Reporting Items for Systematic Reviews and Meta-Analyses guidelines (PRISMA; 24) for systematic reviews (see **Supplementary 1**). Data generated from this review, including details regarding our inclusion/exclusion decisions, are available at https://osf.io/x4e6f/.

### 2.1 Search strategy

To identify seminal ADHD games in the research literature, we first searched for systematic reviews and meta-analyses using OneSearch. OneSearch is a library search engine that scans dozens of interprofessional databases and local digital library materials simultaneously, while minimizing duplications. Our search phrase specified the diagnosis (“ADHD”), clinical focus (“intervention OR treatment OR training OR therap*”), delivery mechanism (“computer* OR digital* OR technolog*”), format (“game OR gamification”), and study type (“meta-analysis OR systematic review”).^1^ We noted a three-year gap between reviews from 2015 to 2018 (cf. 11), followed by a surge that seemed to mark new interest in ADHD games. As a result, we limited our search to the period from January 1, 2018, to December 31, 2024, to capture the recent spate of systematic reviews and meta-analyses (but did not apply any date restrictions to constituent studies).

The first author screened all candidate reviews using the titles and abstracts and omitted reviews clearly unrelated to our topic. The retained papers were then read by two or more co-authors and selected based on the following inclusion/exclusion criteria: (a) published in a peer-reviewed journal; (b) reports a unique systematic review and/or meta-analysis; (c) focuses specifically on ADHD rather than a range of conditions; and (d) focuses on studies of game-based interventions for children or adolescents. Once a set of suitable reviews was identified, all constituent studies were listed. We then excluded individual studies that (e) appear in only one review; (f) are not an efficacy, effectiveness, nor feasibility study of an intervention; (g) do not involve a computer-delivered game; (h) do not include school-age participants with ADHD; or (i) was not published in a peer-reviewed journal. We focused on repeatedly cited studies (criterion e) to ensure that the publications are seminal within this literature. Citation analysis is a common bibliometric technique for identifying key publications (25), which was vital in this case because game studies often involve exploratory and transitory technologies. By relying on citation across multiple, independent review teams, we also avoided definitional challenges around disputed terms like “serious games” and “gamification” (see 26). Dyads of co-authors then independently read each candidate study and applied the remaining criteria (f-i) to remove irrelevant or non-refereed sources. Disagreements were settled by the first author.

### 2.2 Game content

When necessary, we collected supplemental game descriptions from additional sources referenced in the articles or by searching scholarly databases for product descriptions, game overviews, or secondary publications using the game title as a search term (see **Supplementary 2**). For clarity, games that have undergone title changes were combined and attributed to the most recent title (e.g., *EndeavorRx*). We then coded the specific game elements intended to have clinical impact based on definitions of established assessment and treatment practices. To guide this work, we iteratively developed a codebook that functioned as a review protocol (see **Supplementary 3**). The most challenging step was operationalizing CT tasks. As a starting point, we relied on the review by Molitor and Langberg (27) to identify the task paradigms in the ADHD literature, and then operationally defined the tasks tested most often. When gamified, laboratory tasks are often altered (e.g., moving targets in the Corsi block-tapping paradigm), so in addition to our definitions we listed likely modifications. Game elements asserted to be therapeutic that were unlike our codes were reviewed by the team, compared to the relevant literature, and either added to our codebook or deemed “novel” if no precedent could be identified.

Interrater agreement was assessed using the AC_1_ statistic, given our two-rater design and anticipated marginal heterogeneity (28), as estimated by the irrCAC package (29) in *R* (30). The AC_1_ statistic is a chance-adjusted coefficient that can be interpreted similarly to a generalized kappa, with values > 0.80 indicating strong agreement. We then emailed the corresponding author for each CT study in our review to corroborate our findings, given the challenges defining those elements. We were prepared to update our findings if authors provided a compelling rationale; otherwise, we report our conclusions based on our reading of the published game descriptions (author response rate and feedback described below).

### 2.3 Magnitude of effects

To identify promising game content, we converted outcomes into a standardized metric (Cohen’s *d*). A wide variety of instruments are reported in the ADHD games literature (e.g., CT lab measures, rating scales, actigraphy measures) from a mix of within- and between-subjects designs, but parent ratings of ADHD symptoms are commonly used to measure far transfer. Hence, we focused on parent ADHD ratings and report *d-*family effect sizes where sufficient data were available. In two instances, researchers report parent ratings using the original *Behavior Rating Inventory of Executive Function* (BRIEF; 31) instead of ADHD symptoms. The BRIEF can discriminate between children with inattentive and hyperactive-impulsive symptoms (32), so we included those findings in cases where no ADHD symptom measure was reported.

To compute *d*, we used the tools provided by Lenhard and Lenhard (33). ^2^ For single-group or nonequivalent groups pre-post designs we calculated a repeated measures effect, and for pre-post control designs we calculated the Time × Treatment interaction effect. In some instances, effect sizes were only depicted in graphs, and we used a data extraction tool to inform those calculations (WebPlotDigitizer; 35). In all cases, we focused on groups with ADHD and ignored non-ADHD comparison groups, if included. We limited our calculations to immediate pre-post treatment effects, ignoring follow-up measurement occasions, given the variety of designs and follow-up times in this literature. As part of these efforts, we also assessed the risk of bias specific to parent rating outcomes, using tools provided by Cochrane. Specifically, we used the *Revised Risk of Bias* tool (RoB2; 36) for randomized trials, and the *Risk of Bias in Non-randomized Studies of Interventions* tool (ROBINS-I; 37) for non-randomized studies. RoB2 ratings were supported using spreadsheet applications that apply the standard scoring algorithms, as provided by Cochrane (beta ver. 7). To make the results of the RoB2 and ROBINS-I instruments directly comparable, we relabeled the “serious” and “critical” risk of bias determinations on the latter as “high” risk but provide our original determinations in the **Supplementary Materials**. Following online training on the instruments, the first author rated all studies reporting parent ratings and the last author independently rated a randomly selected subset of articles to assess consistency.

## 3 Results

**Figure 1:**
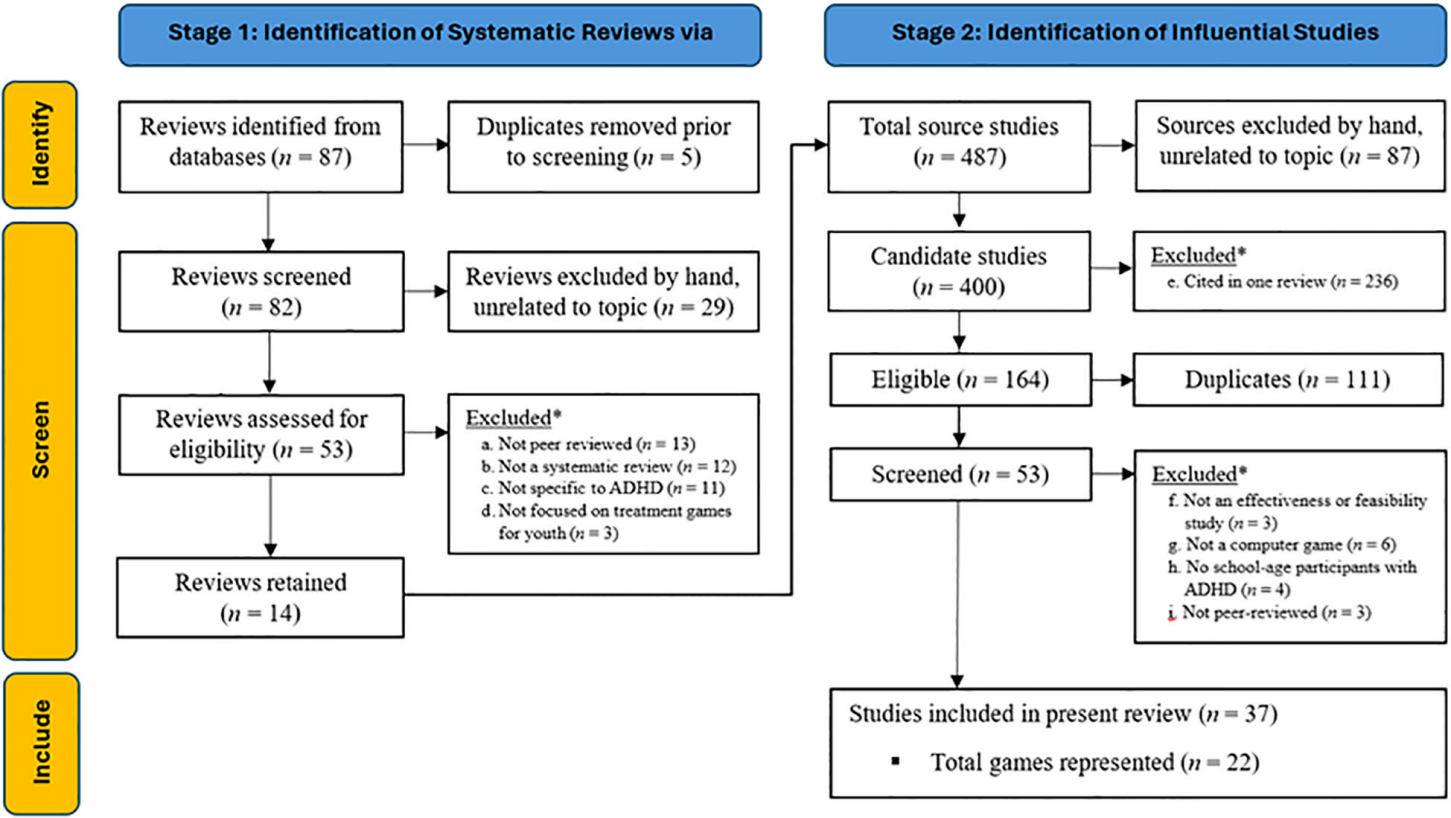
Flow Diagram of Two-stage Literature Review Process to Identify Influential Studies of ADHD Games.

Our search for literature reviews and meta-analyses, last conducted on January 16, 2025, returned 87 publications (see **Figure 1**), with most cross-listed in the Scopus (*n* = 53), PubMed (*n* = 31), and IngentaConnect (*n* = 30) databases. In the title screening process, we were able to exclude five duplicates and another 29 articles that were unavailable in English or clearly unrelated to ADHD games (e.g., focused on other medical conditions, game addiction). We then evaluated the remaining 53 articles using our inclusion/exclusion criteria (a-d) to identify reviews of ADHD games for children and adolescents (exclusion decisions are detailed in **Supplementary 5**). Our efforts identified five meta-analyses (38–42), six systematic reviews (10, 43–47), one scoping review (48), one mapping review (49), and one quasi-systematic review (50), for a total of 14 reviews reporting on 487 partially overlapping studies. The systematic review by Zheng and colleagues (47) appears in arXiv, an online repository for preprint manuscripts and was not peer-reviewed, but we included this manuscript because it appeared to be high-quality, potentially publishable, and already cited 29 times at the time of our review.

We were able to remove 87 constituent studies based on reviewers’ descriptions; specifically, Rodrigo-Yanguas and colleagues’ (50) “quasi-systematic” review included expert opinion papers, narrative texts, case reports, and review articles that we omitted (and that did not overlap the other reviews). In the remaining 400 candidate studies, we excluded another 236 sources that were only cited in a single review (criterion e). We then applied our final exclusion criteria (f-i) as shown in **Figure 1** to arrive at 37 repeatedly cited studies, published between February 2005 and March 2021, describing 22 games (51–87; all decisions and reviewer notes are provided in the accompanying dataset).

The games and the source studies are summarized in **Table 1**. For the content analysis, teams of two co-authors working independently coded games using the primary studies identified through our literature review and supplemental sources. The codebook began with 14 operationalized codes, and another seven were added based on follow-up discussions and iterative feedback from the coders. The coders were largely unfamiliar with neurocognitive assessment and relied solely on the codebook for their determinations. Initial calibration efforts with a subsample of four games resulted in an unacceptable range of interrater reliability estimates (AC_1_ = 0.68 to 1.00). In response, we revised the operational definitions that led to disagreement by adding clarifying language and links to online video demonstrations of the neurocognitive tasks. In a third round of coding with the same four games, interrater reliability increased to an acceptable level (AC_1_ = 0.93 to 1.00). We then used that version of the codebook for all games, with any remaining disagreements settled by the first author. Initial coder agreement remained acceptable (AC_1_ = 0.78 to 1.00), and instances of disagreement were resolved in follow-up communications. See **Table 2** for our final content determinations. As part of these efforts, we also assessed interrater agreement for the risk-of-bias analyses. Double coding was completed in a randomly selected subset of eight articles (31%), resulting in an acceptable interrater agreement rate (87.5%), with only one discrepancy that required revised ratings (i.e., overlooked trial registration date).

**Table 1:** Summary of influential game studies for children with ADHD and immediate parent-reported treatment effects.

Game name	Brief description	Primary studies	Design	ADHD sample (*n*)	Ages	Treatment length	Parent scale	Results (*d*)	Overall RoB
*ACTIVATE*	Online program comprised of three unlinked minigames (e.g., “Catch the Ball”) intended to modulate eight cognitive functions, including sustained attention, working memory, and response inhibition.	Bikic et al. (2018) (51)	RCT	Tx = 31 TAU = 34	6-13	8-weeks (32 hrs)	ADHD-RS	IA = 0.53 H/I = 0.35 COMB *=* 0.41	High
*Adaptive Inhibitory Control Training*	A collection of three minigames (e.g., baseball, feed the fish), developed by NeuroScouting LLC, for training inhibitory control via iPad. To control for placebo effects, adaptive versions of the minigames were compared to nonadaptive versions.	Meyer et al. (2020) (52)	RCT	Tx = 20 Placebo = 20	8-11	4-weeks (≈ 4–5 hrs)	SNAP	IA = 0.56 H/I = 0.26	Low
*Adventurous Dreaming Highflying Dragon (adhD)*	A dragon-themed cognitive training game using a motion-reading camera (Xbox Kinect) and the player’s full-body movements, which adds physical activity. The game is comprised of three levels intended to improve inhibitory control.	Weerdmeester (2016) (53)	RCT	Tx = 37 Control = 36	6-13	3-weeks (90 mins)	None	–	–
*AixTent*	A set of four simple minigames that train alertness, vigilance, selective, and divided attention. In this 2011 study, three of the minigames were tested. In recent years, *AixTent* has been further developed and integrated into “CogniPlus.”	Tucha (2011) (54)	RCT	Tx = 16 Control = 16	10-11	4-weeks (8 hrs)	None	–	–
*ATHYNOS*	An augmented-reality game paired with a motion-reading camera (Xbox Kinect) to address dyspraxia in children with ADHD. The game uses characters familiar to children in Ecuador in minigames, or “scenes,” meant to improve motor skills (e.g., hand-eye coordination).	Avila-Pesantez et al. (2018) (55)	Within subjects	Tx = 11	7-10	4-weeks (80 mins)	None	–	–
*Bio Trace+*	A series of three neurofeedback minigames that respond to, and reward, successful concentration (decreased theta/beta ratio). *Bio Trace+* was compared to electro-myography biofeedback to control for the effect of immediate game feedback.	Bakhshayesh et al. (2011) (56)	RCT	Tx = 18 Biofeedback placebo = 17	6-14	10–15 weeks (15 hrs)	ADHD-RS	**IA = .94** H = .51 I = .39 COMB = .77	Some
*Boogies Academy/Cuibrain*	Separate but related mobile games targeted at two age groups and based on the Theory of Multiple Intelligences. Ten minigames target abilities such as visual-spatial, logical-mathematical, and intrapersonal intelligences.	García-Redondo et al. (2019) (57)	RCT	Tx = 24 TAU = 20	6-16	14-weeks (≈ 9 hrs)	EDAH	IA = -0.17 H/I = 0.04 COMB = -0.24	High
*Braingame Brian*	A third-person role playing game where the player invents machines to solve problems in an immersive and expanding game world. The game targets visuospatial working memory, inhibition, and cognitive flexibility.	Dovis et al. (2019) *(follow-up on Dovis* et al.*, 2015)* (58)	RCT	Full Tx = 31 Placebo = 30	8-12	5-weeks (15–21 hrs)	See below	–	–
		Dovis et al. (2015) (59)	RCT	Full Tx = 31 Placebo = 30	8-12	5-weeks (15–21 hrs)	DBD-RS	IA = 0.23 H/I = 0.14	Low
		Prins et al. (2013) *(follow-up on Van der Oord* et al.*, 2012)* (60)	RCT	Tx = 18 Waitlist = 22	8-12	6-weeks (≈ 17 hrs)	See below	–	–
		Van der Oord et al. (2012) (61)	RCT	Tx = 18 Waitlist = 22	8-12	6-weeks (≈ 17 hrs)	DBD-RS	**IA = 1.15** **H/I = 1.06**	High
*Cogmed WMT*	A collection of minigames using robot, space, or world-building themes (for school-age players). The minigames are intended to modulate verbal working memory, visual-spatial working memory, and visual tracking. Cogmed has undergone multiple changes since its inception in 1999, but two versions are most studied in the “influential” studies: One that adapts task difficulty based on player performance (“RoboMemo”) and a less challenging version that does not (“MegaMemo”). The latter has often been used as a placebo (or low dose) comparison condition for the former.	Bigorra et al. (2016) (62)	RCT	Tx = 36 Placebo = 30	7-12	5 weeks (12–19 hrs)	CBRS	COMB = -0.22	High
		Chacko et al. (2014) (63)	RCT	Tx = 44 Placebo = 41	7-11	5-weeks (12–19 hrs)	DBD-RS	IA *= -*0.24 H/I = -0.24	Low
		van Dongen-Boomsma et al. (2014) (64)	RCT	Tx = 27 Placebo = 24	5-7	5-weeks (≈ 6 hrs)	BRIEF	BRI = -0.21 MI = 0.25 GEC = 0.31	Some
		Egeland et al. (2013) (65)	RCT	Tx = 38 TAU = 37	10-12	5–7 weeks (≈ 18 hrs)	ADHD-RS	IA = 0.56 HI = 0.24 COMB = 0.44	High
		Green et al. (2012) (66)	RCT	Tx = 12 Placebo = 14	7-14	>3 weeks (≈ 14 hrs)	CBRS	COMB = 0.21	High
		Klingberg et al. (2005) (67)	RCT	Tx = 20 Placebo = 24	7-12	5-weeks (≈ 17 hrs)	DSM-IV Scale	**IA = 0.88** **H/I = 0.22**	High
*CogoLand*	A third person racing game where players accelerate their character via EEG electrodes measuring beta wave activity. Players respond to added stimuli (fruits) using a keyboard in higher levels. The game targets attention and concentration.	Lim et al. (2019) (68)	RCT	Tx = 81 Waitlist = 82	6-12	8-weeks (12 hrs)	ADHD-RS	**IA = 0.49**	High
		Lim et al. (2012) (69)	Within subjects	Tx = 19	6-12	8-weeks (12 hrs)	ADHD-RS	**IA *=* 0.78** **H/I = 0.84** **COMB = 0.85**	High
*Computerized Progressive Attentional Training (CPAT)*	CPAT is a progressive attentional training program comprised of four tasks targeting sustained attention, selective attention, orienting attention, and executive attention. The tasks are gamified using simple graphics (e.g., cars for target stimuli).	Shalev et al. (2007) (70)	RCT	Tx = 20 Control = 16	6-13	8-weeks (16 hrs)	ADHD-RS	**IA = 0.99** H/I = 0.53	High
*EndeavorRx*	A third-person, alien-themed racing game where players collect or avoid target stimuli, with algorithmically adjusted difficulty. The game targets attention and inhibitory control. Formerly named *AKL-T01* and *Project EVO.*	Kollins et al. (2021) ^†^ ^(^ 71)	Pre-Post unequal groups	Meds = 124 No meds = 71	8-14	12-weeks (≈ 16 hrs)	ADHD-RS	**IA = 0.74** **H/I = 0.68** **COMB = 0.79**	High
		Kollins et al. (2020) (72)	RCT	Tx = 173 Control = 164	8-12	4-weeks (≈ 8 hrs)	ADHD-RS	IA = 0.11 H/I = -0.55 COMB = 0.07	Low
		Davis et al. (2018) (73)	Pre-Post matched-groups	Tx = 40 (comparison group no ADHD)	8-12	4-weeks (10–15 hrs)	None	–	–
*Eye-contact Training Game*	The eye-contact training game (otherwise unnamed) uses a mixed reality head-mounted display to help children with ADHD focus on faces for increasing periods of time. The aim of the intervention is to improve facial recognition, a critical element of social interaction.	Kim et al. (2020) (74)	RCT^††^	Tx = 20 Control = 20	<11	6-weeks (≈ 8 hrs)	None	–	–
*Focus Pocus*	A suite of tablet-based minigames using a wizard-training theme that provides cognitive training and neurofeedback (using a specialized EEG headset). The game targets impulse control, working memory, and attention. Two earlier studies (i.e., Johnstone et al., 2010; 2012) informed *Focus Pocus*, testing minigames (e.g., “Feed the Monkey,” “Go Go No-go”), that were sometimes paired with attention monitoring (AM) via a single-channel EEG device.	Johnstone et al. (2017) (75)	RCT	Tx = 22 Waitlist = 22 (also 41 subclinical cases)	7-12	7-8-weeks (≈ 8 hrs)	ADHD-RS	COMB = 1.12	High
		Johnstone et al. (2012) (76)	RCT	Tx = 22 Tx + AM = 18 Waitlist = 20 (also compared to 68 typical cases)	7-13	5-weeks (≈ 6–8 hrs)	DSM-IV Scale	COMB = 1.26	High
		Johnstone et al. (2010) (77)	RCT	Tx = 18 Control = 20	7-12	5-weeks (≈ 8 hrs)	DSM-IV Scale	COMB = 0.14	High
*HappyNeuron Pro*	A collection of minigames targeting sustained attention, working memory, and visuospatial memory. Formerly named *Scientific Brain Training* (SBT). (Our content analysis focuses on the six SBT minigames tested by Bikic et al., 2017)	Bikic et al. (2017) (78)	RCT	Tx = 9 Control = 8	14-17	7-weeks (≈ 18 hrs)	ADHD-RS	COMB = 0.29	Some
*Integrated Brain, Body, & Social Intervention (IBBS)*	A combination of computer games, physical exercises, and group behavior contingencies to improve ADHD symptoms. The computer games were based on *ACTIVATE* (above), and targeted cognitive abilities like sustained attention, response inhibition, and working memory.	Smith et al. (2020) (79)	RCT	Tx = 48 Waitlist = 44	5-9	15-weeks (20 hrs)	SNAP	COMB = 0.21	High
*N-back Training*	A computerized, spatial *n*-back task with minimal gamification (i.e., “game-like”), varying difficulty (i.e., levels), and a point system tied to real-world rewards.	Jones et al. (2020) (80)	RCT	Tx = 41 Control = 39	7-14	5-weeks (5 hrs)	CBRS	COMB = 0.27	Some
*Plan-It Commander*	A role-playing space-themed game where players are given missions to mine rare space minerals. The game targets time management, planning, organization, and prosocial skills.	Bul et al. (2018) *(follow-up on Bul* et al.*, 2016)* (81)	Random crossover study	Group 1 = 88 Group 2 = 82	8-12	10-weeks (up to 33 hrs)	See below	–	–
		Bul et al. (2016) (82)	Random crossover study	Group 1 = 88 Group 2 = 82	8-12	10-weeks (up to 33 hrs)	BRIEF	**Plan/Org = 0.22** WM = 0.16	High
		Bul et al. (2015) (83)	Pre-Post	Tx = 42	8-11	8-weeks (≈ 14 hrs)	None	–	–
*RECOGNeyes*	A novel eye-tracking game to treat ADHD, based on the connection between attention and gaze direction. In the game, eye gaze is used to catch snowflakes while avoiding fire. A comparison group played the same game using a computer mouse rather than the eye tracker.	García-Boas et al. (2019) (84)	RCT	Tx = 14 Control = 14	8-15	3-weeks (9 hrs)	None	–	–
*Shape Up*	An exercise game developed commercially for XBOX Kinect. Players are guided through multiple physical exercises (e.g., pushups, squats), and their physical movements are integrated into onscreen, competitive minigames.	Benzing & Schmidt (2019) (85)	RCT	Tx = 28 Waitlist = 23	8-12	8-weeks (12 hrs)	CBRS	IA = 0.35 HI = 0.27 COMB = 0.32	Some
*SmartMind*	A neurofeedback and cognitive training game targeting inhibitory control and working memory through sports-like games that respond to the players’ theta and beta wave activity.	Rajabi et al. (2020) (86)	RCT	Tx = 16 Waitlist = 16	9-11	12-weeks (≈ 22 hrs)	CBRS	**IA = 0.80** **HI = 0.40**	High
*Supermecha* ^†††^	A gamified working memory trainer using a unique game narrative. The player must save villages from evil robots by accurately recalling sequences of visually presented stimuli.	Prins et al. (2011) (87)	RCT	Tx = 27 Control = 24	7-12	3-weeks (45–105 mins)	None	–	–

“Primary studies” appear in two or more systematic reviews/meta-analyses, and references are provided below. Treatment length is reported for the *intended* computer-based component of the program and may have fallen short of the target in some instances. All effect size estimates (*d*) were estimated using the calculators provided by Lenhard & Lenhard (33), and outcomes reported as statistically significant are bolded. Positive effect sizes show an advantage to the treatment, whereas negative effect sizes suggest an advantage to the comparison condition.

ADHD-RS, Attention Deficit/Hyperactivity Rating Scale (88); AI, artificial intelligence; BRIEF, The Behavior Rating Inventory of Executive Function (89); BRI, BRIEF behavioral regulation index; CBRS, Connors Comprehensive Behavior Rating Scales (90); CT, cognitive training; COMB, combined or total ADHD score; DBD-RS, Disruptive Behavior Disorders Rating Scale (91); DSM-IV Scale, any bespoke rating scale derived from the Diagnostic and Statistical Manual of Mental Disorders, Fourth Edition (92); EDAH, Evaluation of the Deficit of Attention and Hyperactivity scale (93); EEG, electroencephalogram; GEC, BRIEF global executive composition; H/I, Hyperactivity/Impulsivity score; IA, Inattention score; MI, BRIEF metacognition index; NF, neurofeedback; RoB, risk of bias as measured by the RoB2 or ROBINS-I tools; SNAP, Swanson, Nolan, and Pelham Scale (94); WM, Working Memory.

^†^ For this study, we report the within-subjects pre-post effect size for the entire sample because a comparison across medication statuses was not intended by the authors.

^††^ We assume this study was randomized, but the authors do not explicitly state how group assignment was conducted.

^†††^ *Supermecha* is unnamed in the original study, but other sources refer to it by this title.

**Table 2:** Content analysis of the games included in the present review.

Game Title	CT													NF	BT	Novel
	CB	CF	Cnc	CPT	DL	DS	FT	GNG	NB	SC	SS	Strp	WCS			
ACTIVATE*	–	–	–	**□**	–	–	–	**□**	–	–	–	–	–	–	–	–
Adaptive ICT*	–	–	–	–	–	–	–	–	–	–	**✓**	–	–	–	–	–
adhD*	–	–	–	–	–	–	–	**✓**	–	–	–	–	–	–	–	–
*AixTent*	–	–	–	**✓**	**✓**	–	–	**✓**	–	–	–	–	–	–	–	–
*ATHYNOS*	–	–	–	–	–	–	–	–	–	–	–	–	–	–	–	**✓**
*BioTrace+*	–	–	–	–	–	–	–	–	–	–	–	–	–	**✓**	–	–
*Boogies Academy/Cuibrain*	–	–	–	–	–	–	–	–	–	–	–	–	–	–	–	**✓**
Braingame Brian*	**✓**	–	–	–	–	–	–	–	–	–	**✓**	–	**☑**	–	–	–
*Cogmed WMT*	**✓**	–	–	–	–	**✓**	–	–	–	–	–	–	–	–	–	–
*CogoLand*	–	–	–	–	–	–	–	–	–	–	–	–	–	**✓**	–	–
*CPAT*	–	–	**✓**	**✓**	–	–	**✓**	–	–	–	–	**✓**	–	–	–	–
*EndeavorRx*	–	–	–	**✓**	–	–	–	**✓**	–	–	–	–	–	–	–	–
*Eye-contact Training Game*	–	–	–	–	–	–	–	–	–	–	–	–	–	–	–	**✓**
Focus Pocus*	–	–	–	–	–	**✓**	–	**✓**	–	–	–	–	–	**✓**	–	–
HappyNeuron Pro*	**✓**	**✓**	–	–	–	–	–	–	–	**✓**	–	–	–	–	–	–
IBBS*	–	–	–	**□**	–	–	–	**□**	–	–	–	–	**□**	–	–	**✓**
N-back Training*	–	–	–	–	–	–	–	–	**✓**	–	–	–	–	–	–	–
*Plan-It Commander*	–	–	–	–	–	–	–	–	–	–	–	–	–	–	**✓**	**-**
*RECOGNeyes*	–	–	–	–	–	–	–	–	–	–	–	–	–	–	–	**✓**
*Shape Up*	–	–	–	–	–	–	–	–	–	–	–	–	–	–	–	**✓**
*SmartMind*	–	–	–	–	–	–	–	–	–	–	–	–	–	**✓**	–	–
Supermecha*	**✓**	–	–	–	–	–	–	–	–	–	–	–	–	–	–	–

Cognitive training (CT), neurofeedback (NF), and behavior therapy (BT) are operationalized in **Supplementary 2**. Novel content met none of the previous categories and appeared unprecedented prior to gamification. Decisions supported by feedback from corresponding authors are marked with an asterisk (*). Cells marked with a boxed checkmark (☑) were added to this table following personal communication, and cells marked with an empty box (◻) were noted as uncorroborated following personal communication.

Adaptive ICT, Adaptive Inhibitory Control Training; adhD., Adventurous Dreaming Highflying Dragon; CPAT, Computerized Progressive Attentional Training; IBBS, Integrated Brain, Body, & Social Intervention; CB, Corsi block-tapping task; CF, Complex figure task; Cnc, Cancellation task; CPT, Continuous performance task; DL, Dreary-Liewald task; DS, Digit span task; FT, Flanker task; GNG, Go/no-go task; NB, *n*-back task; SC, Stockings of Cambridge task; SS, Stop signal task; Strp, Stroop task; WCS, Wisconsin card sorting task.

As anticipated, most games (55%) in our sample provide at least one element of CT. Among those games, there are one to four recognizable neurocognitive tasks found in the assessment literature, but there is no single task used in most or all games. Rather, a plurality of CT games deploys variations of the go/no-go, continuous performance, and Corsi block-tapping task paradigms. Another four games (18%) attempt neurofeedback (including one combined with CT), and one game (5%) attempts behavior therapy. Six games (27%) deliver one or more novel elements, including one that targets dyspraxia (ATHYNOS), one game based on the theory of multiple intelligences (Boogie’s Academy/Cuibrain), three games that deliver physical exercise as a primary or secondary element (e.g., Shape Up), and two that are intended to improve eye gaze direction (RECOGNeyes) or facial recognition (Eye-contact Training Game).

We emailed our findings to corresponding authors for the 12 CT games and received feedback for nine of the games (75% response rate). This personal communication led to us adding one game element and recording five other category decisions as uncorroborated (see **Table 2**). Specifically, we added a game element in one instance (Braingame Brian) where the corresponding author provided unpublished design materials providing additional game detail. In the other instances, a developer responsible for two games (ACTIVATE and IBBS) disagreed with our characterization of their game elements, despite consensus among our coders. We report those elements as uncorroborated in deference to the developer’s feedback. Otherwise, all responding authors agreed with our conclusions. Note that these final determinations are reflected in the descriptive statistics reported above.

**Figure 2:**
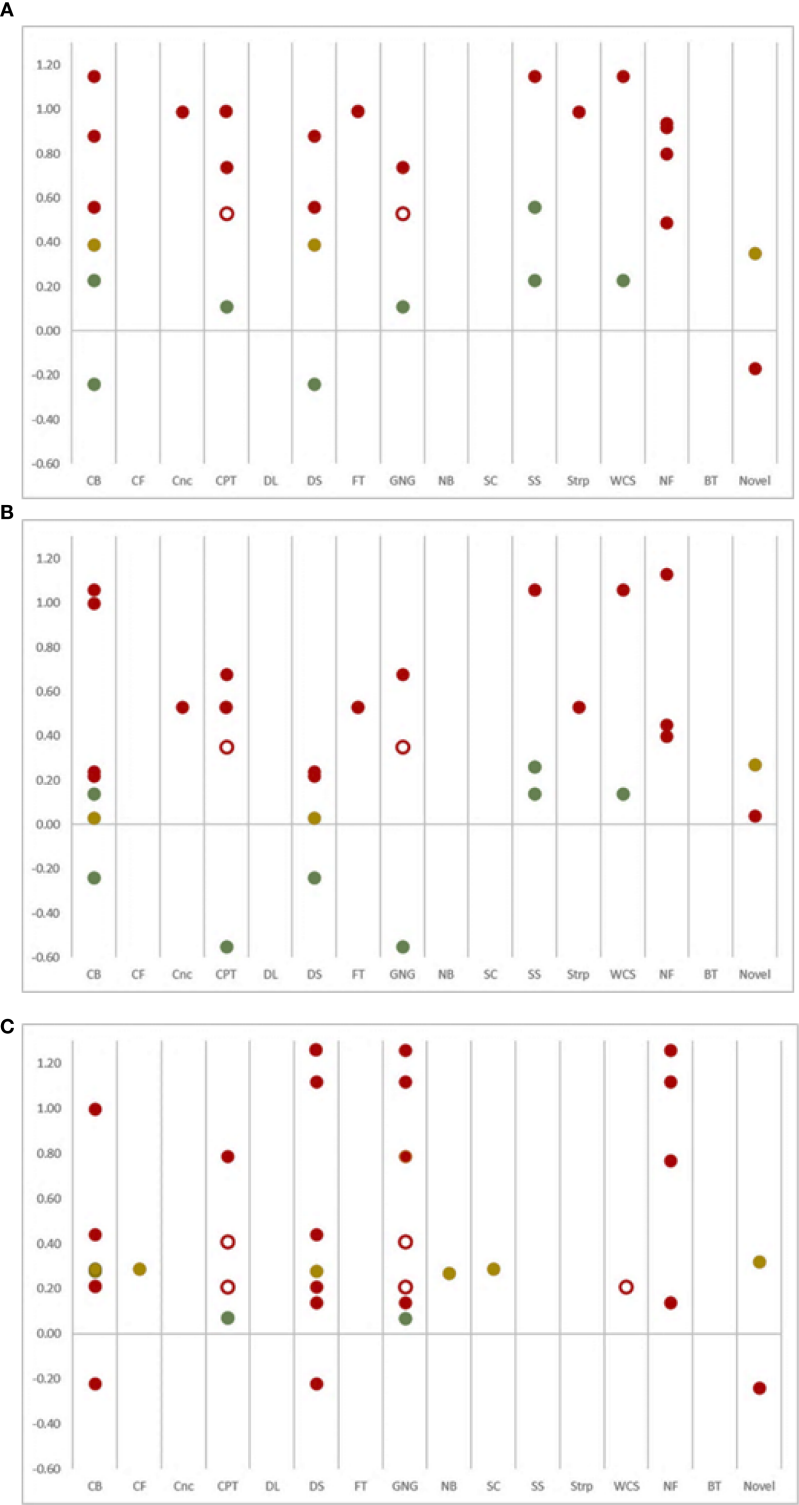
Bubble Plots of Effect Sizes for **(A)** Inattention, **(B)** Hyperactivity-Impulsivity, and **(C)** Combined ADHD Symptoms as Rated by Parents, by Game Content from Low Risk (Green), Some Risk (Yellow) and High Risk (Red) results. Category determinations that were not corroborated by the corresponding authors are indicated with empty bubbles. CB, Corsi block-tapping task; CF, Complex figure task; Cnc, Cancellation task; CPT, Continuous performance task; DL, Dreary-Liewald task; DS, Digit span task; FT, Flanker task; GNG, Go/no-go task; NB, n-back task; SC, Stockings of Cambridge task; SS, Stop signal task; Strp, Stroop task; WCS, Wisconsin card sorting task; NF, Neurofeedback; BT, Behavior therapy.

We then calculated effect size estimates (*d*) of change in parent ratings of ADHD symptoms, where available. Parent ratings were often identified as a primary or secondary outcome, and sufficient data or graphs were provided to estimate at least one effect size for 16 of the 22 games. Estimates range from -0.55 to 1.26. We then examined the relationships between game content and effect sizes on parent ratings of ADHD symptoms. Given the number of unique game elements identified, there was limited data for each. For this reason, our synthesis strategy was to summarize effect size estimate and display those results in a bubble plot (**Figure 2**), consistent with recommendations by McKenzie and Brennan (95). The result does not suggest clear relationships between game content and positive far transfer effects. Instead, the largest effects seem to be associated with the highest risk of bias (for full risk of bias result, see **Supplementary 6**). The risk was most often due to the parent raters’ awareness of their child’s treatment condition (e.g., Domain 4 of the RoB2). No effects from low-risk assessments exceeded *d* = 0.56, with a general pattern of the largest effects associated with the highest risk of bias. Although no game elements were clearly superior, the most promising may be the stop signal task paradigm, based on two low-risk of bias RCTs (*d* range = 0.14 to 0.56).

## 4 Discussion

We identified 22 ADHD games that have appeared in two or more recent systematic reviews or meta-analyses and examined the therapeutic content. Consistent with an experimental therapeutics approach, we were interested in categorizing intervention elements and identifying which are associated with the largest changes in child behavior. We found that ADHD game elements vary widely, even among games with similar objectives (e.g., improved working memory). For example, we uncovered 13 separate cognitive training (CT) mechanisms, with pluralities of CT games deploying content based on the go/no-go, continuous performance, and Corsi block tapping task paradigms. Those tasks ostensibly target sustained attention/response inhibition and visuospatial working memory. But even when successful on lab measures (i.e., near transfer) there is no guarantee these interventions lead to improved behavior elsewhere (i.e., far transfer). So, we examined the relationship between individual game elements and parent ratings of ADHD symptoms.

Our analysis extends previous reviews by showing that no individual game component is clearly superior to any other. In other words, no obvious pattern emerged between the purported mechanisms of action in ADHD games and far transfer outcomes, especially when considering possible sources of bias as depicted in **Figure 2**. Rater expectation bias is a clear confound in this literature that is difficult to avoid in common study designs (e.g., waitlist control); parent raters are often aware of their child’s treatment allocation (i.e., unblinded), which raises the risk assessment to high when using standard risk-of-bias scoring algorithms (see 36). Consistent with expectation bias, effect sizes in the present study tended to be relatively small in the few studies that blinded parent raters to treatment condition, and none were statistically significant. In other instances, studies suffered from attrition problems (e.g., children refused to play the game), which also raises the risk of bias (e.g., Domain 3 of the RoB2). In the end, there are too few high-quality studies using trustworthy far-transfer evaluations to draw definitive conclusions about the ADHD games literature. The available data suggest that bias likely explains those instances when statistically significant and meaningful results are reported. Importantly, this pattern holds true regardless of game content, and no element used in seminal games has demonstrated convincing far transfer effects. It is also critical to note that our data synthesis method does not account for study size, and one potentially promising finding for the *stop signal* task paradigm is based on only 101 study participants.

### 4.1 Why is far transfer so elusive?

As mentioned previously, there are several hypotheses for why ADHD games fail to achieve far transfer. Our results suggest that at least two of these hypotheses are unlikely. First, the hypothesis that each tool only benefits specific subpopulations of individuals with ADHD is partially unsupported because the same pattern of findings emerged for inattentive, hyperactive-impulsive, and combined presentation domains. But our results do not speak to subpopulations defined by other factors (e.g., working memory deficits), so that possibility is still plausible. Second, the hypothesis that ADHD games do not effectively target the core processes impaired in ADHD (i.e., misspecification) is also challenged because we found a wide range of attempted elements with no clear winners; rather, all results were unconvincing. Misspecification remains plausible only if interventions targeting the correct core processes have yet to be tested, published, and reviewed. To completely rule out this possibility, high quality studies with trustworthy measures of far transfer (e.g., blinded parent and/or teacher ratings) are still needed.

We believe the most likely explanation for why ADHD games fail to achieve far transfer is that neurocognitive deficits—the target for most of the games in the present review—are irrelevant to clinical outcomes (i.e., correlation ≠ causation) (18). If true, the most promising future direction is game content based on established psychosocial research. For example, behavior therapy and training interventions are the most established non-medicinal intervention domain, yet only one game in our review, Plan-It Commander (82, 83), delivers behavior interventions. In this case, parent ratings of executive functions suggested significant improvements and small effects on planning-organization and working memory (*d*s = 0.16 to 0.22), albeit with high risk of bias. More research is needed to determine whether serious games targeted to these domains can be effective in schools, but planning and organization interventions are well established in the treatment literature (5).

### 4.2 Limitations

There are several limitations to consider when interpreting our results. First, the studies captured in this review are not exhaustive of the ADHD games literature and do not reflect all games marketed directly to schools and families. Our conclusions are limited to those games that have been studied and then reviewed multiple times in the scholarly literature prior to 2025. Second, we did not play most of the games under investigation and may have miscategorized content based on our reading of the published literature. We tried to avoid error by contacting the authors of the CT studies in our review to verify our conclusions, but not all responded (75% response rate), perhaps due in part to language barriers. Third, we calculated effect size estimates for parent ratings of ADHD symptoms, given the clinical relevance and widespread use of those measures, but these data are not necessarily the best way to judge ADHD game efficacy. Multimethod, multisource measures of ADHD-related impairment would be far more informative, but unfortunately impairment measures are rare in this literature. Readers should also note that *d* estimates for within-subjects designs tend to be larger than for between-subject designs; for this reason, readers are cautioned to also consider statistical significance (bolded in **Table 1**) and the risk of bias when considering our results. Fourth, all neurofeedback games in the present review use some version of theta/beta ratio feedback, but other neurofeedback protocols exist (e.g., sensorimotor rhythm training, slow cortical potential training) that were not captured in the current overview. And fifth, we caution readers not to confuse our use of the term “novel” with “ineffective.” Game elements not appearing in the assessment or treatment literatures may indeed hold promise as interventions for ADHD. Similarly, game design elements, including game mechanics, narratives, and user experiences, might also influence user outcomes, but those factors were beyond the scope of this review.

### 4.3 Recommendations

To our knowledge, this is the first attempt to document and assess the potential promise of specific ADHD game elements. Based on our findings, we offer three recommendations to advance the research and development of these tools. First, content reporting standards would greatly advance this field and render coding efforts, like those attempted here, unnecessary. The lack of reporting standards is particularly problematic in the case of CT games. It is critical that developers cite the laboratory tasks or other source materials that inspire their training elements. Some authors report these details, albeit in companion articles or **Supplementary Materials**, whereas others offer few clues—even in widely cited, peer-reviewed studies. Likewise, novel therapeutic elements must be identified as such, ideally with a rationale for why developers believe this innovation is warranted and how it is intended to work. Therapeutically inert content meant to entertain and motivate players might be identified as “recreational” to avoid confusion with active elements. In our view, purely recreational content that thematically connects therapeutic elements may be necessary to enhance the appeal of ADHD games and engage otherwise reticent children and adolescents.

Second, the ADHD games reviewed here only included clinician support in a few cases. In fact, most games appear to have been developed as standalone interventions, available directly to consumers without the support of coaches, clinicians, trainers, or physicians. Based on our work with adolescents with ADHD, we believe this is impractical. We recommend that any games intended to change behavior are supported by real-world interventionists who coach players as they attempt new skills in their daily lives. Just as homework assignments are common in modern psychotherapies, children provided with a serious game must try the skills trained in the game in non-game settings, ideally structured and monitored by an adult. Game developers might consider parent, teacher, or clinician treatment manuals, and possibly player workbooks, that describe the game and prescribe activities to support transfer of learning. Planned transfer activities might address some of the difficulties to achieve far transfer effects (see 96), but this is an empirical question that has yet to be addressed. We believe this is particularly critical for games targeted to children with ADHD, given the chronic, neurodevelopmental nature of the disorder. But ADHD game design to date appears to be largely driven by market concerns, with priority given to self-contained and readily downloadable products. It would be informative to compare ADHD games across delivery models, where products are either delivered as standalone interventions or as part of a broader intervention package with real-world adult support.

Finally, behavior therapy and skill training interventions appear to be underrepresented in the ADHD games literature and warrant additional research. Traditional behavior therapy is strongly supported in the research literature, and successful efforts to gamify those approaches could expand treatments in homes, schools, and clinics. It is unclear whether games focused on training skills (e.g., organization strategies) could achieve replicable far transfer effects, but this domain may be ripe for development, given the growing literature on training interventions and what seems like a clear potential for gamifying those strategies. But until far transfer is convincingly demonstrated on trustworthy measures of ADHD-related impairments (e.g., organization, classroom performance) across multiple randomized control trials, we cannot safely conclude that any ADHD game—regardless of its content—is an effective treatment option.

## Footnotes

1 We used wildcard characters (*) to capture common variations like “digital heath interventions.” We did not use wildcard characters with the search terms “game” and “gamification” to avoid capturing classroom and other real-world activities, like token economies, that have been referred to loosely as “gamified.” We also avoided variations of “ADHD” to exclude antiquated conceptions of the disorder (e.g., “hyperkinetic disorder”); but this only affected the search for systematic reviews/meta-analyses, not the constituent studies used for data synthesis.

2 To calculate *d*, we relied on the online calculators provided by Lenhard & Lenhard (33). Most often we used the calculator based on the formula attributed to Morris (2008) for pretest-posttest control group designs, which is sometimes denoted as *d*_ppc2_. Alternatively, when eta squared (η^2^) was reported, we used Lenhard and Lenhard’s online calculator to directly transform to Cohen’s *d*. In other instances when standard deviation (*SD*) was provided for two groups or two measurement occasions separately without an effect size estimate, we used Lenhard and Lenhard’s online calculator to estimate pooled SD and added that into the standard formula for Cohen’s *d* (often denoted as *d*_s_). When only partial eta squared ( ${\text{η}}_{\text{p}}^{2}$ ) was provided, we converted to Cohen’s *f*, and then multiplied *f* × 2 to get an estimate of Cohen’s *d* (34). Note that this approach was used in only one instance, where two groups of equal sizes were compared (Johnstone et al., 2017).

## References

[1] Cheng VWS Davenport T Johnson D Vella K Hickie IB. Gamification in apps and technologies for improving mental health and well-being: Systematic review. JMIR Ment Health. (2019) 6:e13717. doi: 10.2196/1371, PMID: 31244479 PMC6617915

[2] Fitzgerald M Ratcliffe G. Serious games, gamification, and serious mental illness: A scoping review. Psychiatr Serv. (2020) 71:170–83. doi: 10.1176/appi.ps.201800567, PMID: 31640521

[3] Aschbrenner KA Naslund JA Tomlinson EF Kinney A Pratt SI Brunette MF. Adolescents’ use of digital technologies and preferences for mobile health coaching in public mental health settings. Front Public Health. (2019) 7:178. doi: 10.3389/fpubh.2019.00178, PMID: 31312629 PMC6614191

[4] Evans SW Beauchaine TP Chronis-Tuscano A Becker SP Chacko A Gallagher R. The efficacy of cognitive videogame training for ADHD and what FDA clearance means for clinicians. Evid Based Pract Child Adolesc Ment Health. (2021) 6:116–30. doi: 10.1080/23794925.2020.1859960

[5] Fabiano GA Schatz NK Aloe AM Pelham WE Smyth AC Zhan Z. Comprehensive meta-analysis of attention-deficit/hyperactivity disorder psychosocial treatments investigated within between group studies. Rev Educ Res. (2021) 91:718–60. doi: 10.3102/00346543211025092

[6] Powell L Parker J Robertson N Harpin V. Attention deficit hyperactivity disorder: Is there an app for that? Suitability assessment of apps for children and young people with ADHD. JMIR mHealth uHealth. (2017) 5:e145. doi: 10.2196/mhealth.7371, PMID: 28978497 PMC5647456

[7] Wolraich ML Hagan JF Allan C Chan E Davison D Earls M. Clinical practice guideline for the diagnosis, evaluation, and treatment of attention-deficit/hyperactivity disorder in children and adolescents. Pediatrics. (2019) 144:e20192528. doi: 10.1542/peds.2019-2528, PMID: 31570648 PMC7067282

[8] Barbaresi WJ Campbell L Diekroger EA Froehlich TE Liu YH O’Malley E. The Society for Developmental and Behavioral Pediatrics clinical practice guideline for the assessment and treatment of children and adolescents with complex Attention-Deficit/Hyperactivity Disorder: Process of care algorithms. J Dev Behav Pediatr. (2020) 41:S58–74. doi: 10.1097/DBP.0000000000000781, PMID: 31996578

[9] Evans SW Owens JS Bunford N. Evidence-based psychosocial treatments for children and adolescents with attention-deficit/hyperactivity disorder. J Clin Child Adolesc Psychol. (2018) 43:527–51. doi: 10.1080/15374416.2013.850700, PMID: 24245813 PMC4025987

[10] Peñuelas-Calvo I Jiang-Lin LK Girela-Serrano B Delgado-Gomez D Navarro-Jimenez R Baca-Garcia E. Video games for the assessment and treatment of attention-deficit/hyperactivity disorder: a systematic review. Eur Child Adolesc Psychiatry. (2022) 31:5–20. doi: 10.1007/s00787-020-01557-w, PMID: 32424511

[11] Rivero TS Herrera Nunez LM Pires EU Amodeo Bueno OF. ADHD rehabilitation through video gaming: A systematic review using PRISMA guidelines of the current findings and the associated risk of bias. Front Psychiatry. (2015) 6:151. doi: 10.3389/fpsyt.2015.00151, PMID: 26557098 PMC4614280

[12] Pievsky MA McGrath RE. The neurocognitive profile of attention-deficit/hyperactivity disorder: A review of meta-analyses. Arch Clin Neuropsychol. (2018) 33:143–57. doi: 10.1093/arclin/acx055, PMID: 29106438

[13] Raz N Lindenberger U. Life-span plasticity of the brain and cognition: From questions to evidence and back. Neurosci Biobehav Rev. (2013) 37:2195–200. doi: 10.1016/jneubeorev.2013.10.003, PMID: 24140011

[14] Schmiedek F. Methods and designs. In: Strobach T Karbach J, editors. Cognitive training: An overview of features and applications, 2nd ed. Berlin: Springer (2016). p. 11–22. doi: 10.1007/978-3-030-39292-5

[15] Rubia K. Neurotherapeutics for ADHD: Do they work? Psych J. (2022) 11:419–27. doi: 10.1002/pchj.544, PMID: 35359026 PMC10083951

[16] Simons DJ Boot WR Charness N Gathercole SE Chabris CF Hambrick DZ. Do “brain training” programs work? Psychol Sci Public Interest. (2016) 17:103–86. doi: 10.1177/1529100616661983, PMID: 27697851

[17] Redick TS. The hype cycle of working memory training. Curr Dir Psychol Sci. (2019) 28:423–9. doi: 10.1177/09637214198486, PMID: 31814661 PMC6897530

[18] Smid CR Karbach J Steinbeis N. Toward a science of effective cognitive training. Curr Dir Psychol Sci. (2020) 29:531–7. doi: 10.1177/0963721420951599

[19] Kofler MJ Irwin LN Soto EF Groves NB Harmon SL Sarver DE. Executive functioning heterogeneity in pediatric ADHD. J Abnorm Child Psychol. (2019) 47:273–86. doi: 10.1007/s10802-018-0438-2, PMID: 29705926 PMC6204311

[20] Rapport MD Orban SA Kofler MJ Friedman LM. Do programs designed to train working memory, other executive functions, and attention benefit children with ADHD? A meta-analytic review of cognitive, academic, and behavioral outcomes. Clin Psychol Rev. (2013) 33:1237–52. doi: 10.1016/j.cpr.2013.08.005, PMID: 24120258

[21] Lee CS Chen T Gao Q Jua C Song R Huang X. The effects of theta/beta-based neurofeedback training on attention in children with attention deficit hyperactivity disorder: A systematic review and meta-analysis. Child Psychiatry Hum Dev. (2022) 54:1577–606. doi: 10.1007/s10578-022-01361-4, PMID: 35471754

[22] Insel TR Gogtay N. National Institute of Mental Health clinical trials: New opportunities, new expectations. JAMA Psychiatry. (2014) 71:745–6. doi: 10.1001/jamapsychiatry.2014.426, PMID: 24806613

[23] Raghavan R Munson MR Le C. Toward an experimental therapeutics approach in human services research. Psychiatr Serv. (2019) 70:1130–7. doi: 10.1176/appi.ps.201800577, PMID: 31500543

[24] Page MJ McKenzie JE Bossuyt PM Boutron I Hoffmann TC Mulrow CD. The PRISMA 2020 statement: An updated guideline for reporting systematic reviews. BMJ. (2021) 372:n71. doi: 10.1136/bmj.n71, PMID: 33782057 PMC8005924

[25] Passas I. Bibliometric analysis: the main steps. Encyclopedia (Basel Switzerland). (2024) 4:1014–25. doi: 10.3390/encyclopedia4020065

[26] Stenros J. The game definition game: A review. Games Cult. (2016) 12:499–520. doi: 10.1177/1555412016655679

[27] Molitor SJ Langberg JM. Using task performance to inform treatment planning for youth with ADHD: A systematic review. Clin Psychol Rev. (2017) 58:157–73. doi: 10.1016/j.cpr.2017.10.007, PMID: 29096908

[28] Gwet KL. Computing inter-rater reliability and its variance in the presence of high agreement. Br J Math Stat Psychol. (2008) 61:29–48. doi: 10.1348/000711006X126600, PMID: 18482474

[29] Gwet KL. irrCAC: Computing chance-corrected agreement coefficients (CAC) (R package version 1.0) [computer software]. The Comprehensive R Archive Network (2019). Available online at: https://cran.r-project.org/src/contrib/irrCAC_1.0.tar.gz (Accessed January 15, 2025).

[30] R Core Team. R: A language and environment for statistical computing. Vienna, Austria: R Foundation for Statistical Computing (2021). Available online at: https://www.R-project.org/ (Accessed January 15, 2025).

[31] Gioia GA Isquith PK Guy SC Kenworthy L. Behavior Rating Inventory of Executive Function (BRIEF) [Database record]. APA PsycTests. (2000). doi: 10.1037/t73087-000

[32] Jacobson LA Pritchard AE Koriakin TA Jones KE Mahone EM. Initial examination of the BRIEF-2 in clinically referred children with and without ADHD symptoms. J Atten Disord. (2020) 24:1775–84. doi: 10.1177/1087054716663632, PMID: 27519529 PMC5303680

[33] Lenhard W Lenhard A. Computation of effect sizes. Psychometrica. (2016). doi: 10.13140/RG.2.2.17823.92329

[34] Cohen J. Statistical power Analysis for the Behavioral Sciences. 2nd ed. New York: Lawrence Erlbaum Associates, Inc (1988).

[35] Rohatgi A. WebPlotDigitizer (Version 5) (2024). Available online at: https://automeris.io/ (Accessed January 15, 2025).

[36] Sterne JAC Savović J Page MJ Elbers RG Blencowe NS Boutron I. RoB 2: A revised tool for assessing risk of bias in randomised trials. BMJ. (2019) 366:l4898. doi: 10.1136/bmj.l4898, PMID: 31462531

[37] Sterne JAC Hernán MA Reeves BC Savović J Berkman ND Viswanathan M. ROBINS-I: A tool for assessing risk of bias in non-randomized studies of interventions. BMJ. (2016) 355:i4919. doi: 10.1136/bmj.i4919, PMID: 27733354 PMC5062054

[38] Oh S Choi J Han DH Kim E. Effects of game-based digital therapeutics on attention deficit hyperactivity disorder in children and adolescents as assessed by parents or teachers: A systematic review and meta-analysis. Eur Child Adolesc Psychiatry. (2024) 33:481–93. doi: 10.1007/s00787-023-02174-z, PMID: 36862162

[39] Qiu H Liang X Wang P Zhang H Shum DHK. Efficacy of non-pharmacological interventions on executive functions in children and adolescents with ADHD: A systematic review and meta-analysis. Asian J Psychiatr. (2023) 87. doi: 10.1016/j.ajp.2023.103692\, PMID: 37450981

[40] Westwood SJ Parlatini V Rubia K Cortese S Sonuga-Barke EJ. Computerized cognitive training in attention-deficit/hyperactivity disorder (ADHD): A meta-analysis of randomized controlled trials with blinded and objective outcomes. Mol Psychiatry. (2023) 28:1402–14. doi: 10.1038/s41380-023-02000-7, PMID: 36977764 PMC10208955

[41] Yu C Wang C Xie Q Wang C. Effect of virtual reality technology on attention and motor ability in children with attention-deficit/hyperactivity disorder: Systematic review and meta-analysis. JMIR Serious Games. (2024) 12:e5691281. doi: 10.2196/56918, PMID: 39602820 PMC11612531

[42] Zhang W Li H Sheng Y. A study of the effects of virtual reality-based sports games on improving executive and cognitive functions in minors with ADHD—A meta-analysis of randomized controlled trials. Behav Sci. (2024) 14:1141. doi: 10.3390/bs14121141, PMID: 39767283 PMC11673233

[43] Caselles-Pina L Sújar A Quesada-López A Delgado-Gómez D. Adherence, frequency, and long-term follow-up of video game-based treatments in patients with attention-deficit/hyperactivity disorder: A systematic review. Brain Behav. (2023) 13:e3265. doi: 10.1002/brb3.3265, PMID: 37743605 PMC10636395

[44] Cervantes JA López S Cervantes S Hernández A Duarte H. Social robots and brain–computer interface video games for dealing with attention deficit hyperactivity disorder: A systematic review. Brain Sci. (2023) 13:1172. doi: 10.3390/brainsci13081172, PMID: 37626528 PMC10452217

[45] Lim CG Lim-Ashworth NS Fung DS. Updates in technology-based interventions for attention deficit hyperactivity disorder. Curr Opin Psychiatry. (2020) 33:577–85. doi: 10.1097/YCO.0000000000000643, PMID: 32858596 PMC7575028

[46] Powell L Parker J Harpin V. What is the level of evidence for the use of currently available technologies in facilitating the self-management of difficulties associated with ADHD in children and young people? A systematic review. Eur Child Adolesc Psychiatry. (2018) 27:1391–412. doi: 10.1007/s00787-017-1092-x, PMID: 29222634

[47] Zheng Y Li R Li S Zhang Y Yang S Ning H. A review on serious games for ADHD. arXiv preprint arXiv:2105.02970. (2021). https://arxiv.org/abs/2105.02970 (Accessed January 15, 2025).

[48] Jiang H Natarajan R Shuy YK Rong L Zhang MW Vallabhajosyula R. The use of mobile games in the management of patients with attention deficit hyperactive disorder: A scoping review. Front Psychiatry. (2022) 13:792402. doi: 10.3389/fpsyt.2022.792402, PMID: 35308884 PMC8931195

[49] Lakes KD Cibrian FL Schuck SE Nelson M Hayes GR. Digital health interventions for youth with ADHD: A mapping review. Comput Hum Behav Rep. (2022) 6:100174. doi: 10.1016/j.chbr.2022.100174

[50] Rodrigo-Yanguas M González-Tardón C Bella-Fernández M Blasco-Fontecilla H. Serious video games: Angels or demons in patients with attention-deficit hyperactivity disorder? A quasi-systematic review. Front Psychiatry. (2022) 13:798480. doi: 10.3389/fpsyt.2022.798480, PMID: 35573357 PMC9091561

[51] Bikic A Leckman JF Christensen TØ Bilenberg N Dalsgaard S. Attention and executive functions computer training for attention-deficit/hyperactivity disorder (ADHD): Results from a randomized, controlled trial. Eur Child Adolesc Psychiatry. (2018) 27:1563–74. doi: 10.1007/s00787-018-1151-y, PMID: 29644473

[52] Meyer KN Santillana R Miller B Clapp W Way M Bridgman-Goines K. Computer-based inhibitory control training in children with Attention-Deficit/Hyperactivity Disorder (ADHD): Evidence for behavioral and neural impact. PloS One. (2020) 15:e0241352. doi: 10.1371/journal.pone.0241352, PMID: 33253237 PMC7703966

[53] Weerdmeester J Cima M Granic I Hashemian Y Gotsis M. A feasibility study on the effectiveness of a full-body videogame intervention for decreasing attention deficit hyperactivity disorder symptoms. Games Health J. (2016) 5:258–69. doi: 10.1089/g4h.2015.0103, PMID: 27304677

[54] Tucha O Tucha L Kaumann G König S Lange KM Stasik D. Training of attention functions in children with attention deficit hyperactivity disorder. ADHD Atten Defic Hyperact Disord. (2011) 3:271–83. doi: 10.1007/s12402-011-0059-x, PMID: 21597880 PMC3158847

[55] Avila-Pesantez D Rivera LA Vaca-Cardenas L Aguayo S Zuñiga L. (2018). Towards the improvement of ADHD children through augmented reality serious games: Preliminary results, in: 2018 IEEE Global Engineering Education Conference (EDUCON), (2018) pp. 843–8. IEEE. doi: 10.1109/EDUCON.2018.8363318

[56] Bakhshayesh AR Hänsch S Wyschkon A Rezai MJ Esser G. Neurofeedback in ADHD: A single-blind randomized controlled trial. Eur Child Adolesc Psychiatry. (2011) 20:481–91. doi: 10.1007/s00787-011-0208-y, PMID: 21842168

[57] García-Redondo P García T Areces D Núñez JC Rodríguez C. Serious games and their effect improving attention in students with learning disabilities. Int J Environ Res Public Health. (2019) 16:2480. doi: 10.3390/ijerph16142480, PMID: 31336804 PMC6679141

[58] Dovis S Maric M Prins PJ van der Oord S. Does executive function capacity moderate the outcome of executive function training in children with ADHD? ADHD Atten Defic Hyperact Disord. (2019) 11:445–60. doi: 10.1007/s12402-019-00308-5, PMID: 31123915

[59] Dovis S van der Oord S Wiers RW Prins PJ. Improving executive functioning in children with ADHD: Training multiple executive functions within the context of a computer game. A randomized double-blind placebo controlled trial. PloS One. (2015) 10:e0121651. doi: 10.1371/journal.pone.0121651, PMID: 25844638 PMC4386826

[60] Prins PJ Brink ET Dovis S Ponsioen A Geurts HM De Vries M. Braingame Brian”: Toward an executive function training program with game elements for children with ADHD and cognitive control problems. Games Health J. (2013) 2:44–9. doi: 10.1089/g4h.2013.0004, PMID: 26196554

[61] Van der Oord S Ponsioen AJ Guerts HM Ten Brink EL Prins PJ. A pilot study of the efficacy of a computerized executive functioning remediation training with game elements for children with ADHD in an outpatient setting: Outcome on parent- and teacher-rated executive functioning and ADHD behavior. J Atten Disord. (2012) 18:699–712. doi: 10.1177/1087054712453167, PMID: 22879577

[62] Bigorra A Garolera M Guijarro S Hervás A. Long-term far-transfer effects of working memory training in children with ADHD: A randomized controlled trial. Eur Child Adolesc Psychiatry. (2016) 25:853–67. doi: 10.1007/s00787-015-0804-3, PMID: 26669692

[63] Chacko A Bedard AC Marks DJ Feirsen N Uderman JZ Chimiklis A. A randomized clinical trial of Cogmed working memory training in school-age children with ADHD: A replication in a diverse sample using a control condition. J Child Psychol Psychiatry. (2014) 55:247–55. doi: 10.1111/jcpp.12146, PMID: 24117656 PMC3944087

[64] van Dongen-Boomsma M Vollebregt MA Buitelaar JK Slaats-Willemse D. Working memory training in young children with ADHD: A randomized placebo-controlled trial. J Child Psychol Psychiatry. (2014) 55:886–96. doi: 10.1111/jcpp.12218, PMID: 24628438

[65] Egeland J Aarlien AK Saunes BK. Few effects of far transfer of working memory training in ADHD: A randomized controlled trial. PloS One. (2013) 8:e75660. doi: 10.1371/journal.pone.0075660, PMID: 24124503 PMC3790857

[66] Green CT Long DL Green D Iosif AM Dixon JF Miller MR. Will working memory training generalize to improve off-task behavior in children with attention-deficit/hyperactivity disorder? Neurotherapeutics. (2012) 9:639–48. doi: 10.1007/s13311-012-0124-y, PMID: 22752960 PMC3441930

[67] Klingberg T Fernell E Olesen PJ Johnson M Gustafsson P Dahlström K. Computerized training of working memory in children with ADHD-a randomized, controlled trial. J Am Acad Child Adolesc Psychiatry. (2005) 44:177–86. doi: 10.1097/00004583-200502000-00010, PMID: 15689731

[68] Lim CG Poh XWW Fung SSD Guan C Bautista D Cheung YB. A randomized controlled trial of a brain-computer interface based attention training program for ADHD. PloS One. (2019) 14:e0216225. doi: 10.1371/journal.pone.0216225, PMID: 31112554 PMC6528992

[69] Lim CG Lee TS Guan C Fung DS Zhao Y Teng SS. A brain-computer interface based attention training program for treating attention deficit hyperactivity disorder. PloS One. (2012) 7:1–8. doi: 10.1371/journal.pone.0046692, PMID: 23115630 PMC3480363

[70] Shalev L Tsal Y Mevorach C. Computerized progressive attentional training (CPAT) program: Effective direct intervention for children with ADHD. Child Neuropsychol. (2007) 13:382–8. doi: 10.1080/09297040600770787, PMID: 17564853

[71] Kollins SH Childress A Heusser AC Lutz J. Effectiveness of a digital therapeutic as adjunct to treatment with medication in pediatric ADHD. NPJ Digit Med. (2021) 4:1–8. doi: 10.1038/s41746-021-00429-0, PMID: 33772095 PMC7997870

[72] Kollins SH DeLoss DJ Cañadas E Lutz J Findling RL Keefe RS. A novel digital intervention for actively reducing severity of paediatric ADHD (STARS-ADHD): A randomised controlled trial. Lancet Digit Health. (2020) 2:e168–78. doi: 10.1016/S2589-7500(20)30017-0, PMID: 33334505

[73] Davis NO Bower J Kollins SH. Proof-of-concept study of an at-home, engaging, digital intervention for pediatric ADHD. PloS One. (2018) 13:e0189749. doi: 10.1371/journal.pone.0189749, PMID: 29324745 PMC5764249

[74] Kim S Ryu J Choi Y Kang Y Li H Kim K. Eye-contact game using mixed reality for the treatment of children with attention deficit hyperactivity disorder. IEEE Access. (2020) 8:45996–6006. doi: 10.1109/ACCESS.2020.2977688

[75] Johnstone SJ Roodenrys SJ Johnson K Bonfield R Bennett SJ. Game-based combined cognitive and neurofeedback training using Focus Pocus reduces symptom severity in children with diagnosed AD/HD and subclinical AD/HD. Int J Psychophysiol. (2017) 116:32–44. doi: 10.1016/j.ijpsycho.2017.02.015, PMID: 28257875

[76] Johnstone SJ Roodenrys S Blackman R Johnston E Loveday K Mantz S. Neurocognitive training for children with and without AD/HD. ADHD Atten Defic Hyperact Disord. (2012) 4:11–23. doi: 10.1007/s12402-011-0069-8, PMID: 22179720

[77] Johnstone SJ Roodenrys S Phillips E Watt AJ Mantz S. A pilot study of combined working memory and inhibition training for children with AD/HD. ADHD Atten Defic Hyperact Disord. (2010) 2:31–42. doi: 10.1007/s12402-009-0017-z, PMID: 21432588

[78] Bikic A Christensen TØ Leckman JF Bilenberg N Dalsgaard S. A double-blind randomized pilot trial comparing computerized cognitive exercises to Tetris in adolescents with attention-deficit/hyperactivity disorder. Nord J Psychiatry. (2017) 71:455–64. doi: 10.1080/08039488.2017.1328070, PMID: 28598701

[79] Smith SD Vitulano LA Katsovich L Li S Moore C Li F. A randomized controlled trial of an integrated brain, body, and social intervention for children with ADHD. J Atten Disord. (2020) 24:780–94. doi: 10.1177/1087054716647490, PMID: 27178060 PMC5107355

[80] Jones MR Katz B Buschkuehl M Jaeggi SM Shah P. Exploring n-back cognitive training for children with ADHD. J Atten Disord. (2020) 24:704–19. doi: 10.1177/1087054718779230, PMID: 29877128 PMC6445784

[81] Bul KC Doove LL Franken IH van der Oord S Kato PM Maras A. A serious game for children with attention deficit hyperactivity disorder: Who benefits the most? PloS One. (2018) 13:e0193681. doi: 10.1371/journal.pone.0193681, PMID: 29543891 PMC5854282

[82] Bul KC Kato PM van der Oord S Danckaerts M Vreeke LJ Willems A. Behavioral outcome effects of serious gaming as an adjunct to treatment of children with attention-deficit/hyperactivity disorder: A randomized controlled trial. J Med Internet Res. (2016) 18:e26. doi: 10.2196/jmir.5173, PMID: 26883052 PMC4773597

[83] Bul KC Franken IH van der Oord S Kato PM Danckaerts M Vreeke LJ. Development and user satisfaction of “Plan-It Commander,” a serious game for ADHD. Games Health J. (2015) 4:502–12. doi: 10.1089/g4h.2015.0021, PMID: 26325247

[84] García-Boas A D’Amelio TD Oliveira I Collins P Echevarria C Zapata LP. Novel interactive eye-tracking game for training attention in children with attention-deficit/hyperactivity disorder. Prim Care Companion CNS Disord. (2019) 21:19m02428. doi: 10.4088/PCC.19m02428, PMID: 31274260

[85] Benzing V Schmidt M. The effect of exergaming on executive functions in children with ADHD: A randomized clinical trial. Scand J Med Sci Sports. (2019) 29:1243–53. doi: 10.1111/sms.13446, PMID: 31050851

[86] Rajabi S Pakize A Moradi N. Effect of combined neurofeedback and game-based cognitive training on the treatment of ADHD: A randomized controlled study. Appl Neuropsychol Child. (2020) 9:193–205. doi: 10.1080/21622965.2018.1556101, PMID: 30734583

[87] Prins PJ Dovis S Ponsioen A Ten Brink E van der Oord S. Does computerized working memory training with game elements enhance motivation and training efficacy in children with ADHD? Cyberpsychol Behav Soc Netw. (2011) 14:115–22. doi: 10.1089/cyber.2009.0206, PMID: 20649448

[88] DuPaul GJ Power TJ Anastopoulos AD Reid R. ADHA Rating Scale-IV: Checklists, norms, and clinical interpretation. New York: Guilford (1998).

[89] Gioia GA Isquith PK Guy SC Kenworthy L. Behavior Rating Inventory of Executive Functions. Lutz: PAR (2000).

[90] Conners CK. Conners 3rd Edition manual. Springer Cham: Multi-Health Systems (2008).

[91] Fosco WD Babinski DE Waschbusch DA. The disruptive behavior disorders rating scale: Updated factor structure, measurement invariance, and national caregiver norms. J Pediatr Psychol. (2023) 48:468–78. doi: 10.1093/jpepsy/jsad006, PMID: 36881692

[92] American Psychiatric Association. Diagnostic and statistical manual of mental disorders. 4th edition. Washington DC: DSM-IV (1994).

[93] Farré A Narbona J. EDAH: Scale for the assessment of attention deficit hyperactivity disorder. Madrid, Spain: TEA Ediciones (2001).

[94] Swanson JM. SNAP-IV Teacher and parent ratings scale. In: Aykr F, editor. Therapist’s Guide to Learning and Attention Disorders. Academic Press (2003). p. 487–500.

[95] McKenzie JE Brennan SE. Chapter 12: Synthesizing and presenting findings using other methods. In: Higgins JPT Thomas J Chandler J Cumpston M Li T Page MJ, editors. Cochrane handbook for systematic reviews of interventions (ver. 6.4). Cochrane (2023). Available online at: www.training.cochrane.org/handbook (Accessed January 15, 2025).

[96] Tamm L Epstein JN Peugh JL Nakonezny PA Hughes CW. Preliminary data suggesting the efficacy of attention training for school-aged children with ADHD. Dev Cognit Neurosci. (2013) 4:16–28. doi: 10.1016/j.dcn.2012.11.004, PMID: 23219490 PMC3617931

