# Supplementary material for: A systematic review and content analysis of serious video games for children with ADHD
Source: Front Psychiatry. 2025 Oct 6;16:1605744. doi: 10.3389/fpsyt.2025.1605744 (PMC12536224; doi:10.3389/fpsyt.2025.1605744)
Supplement: Supplementary file 2 [file Table2.docx]

**Supplemental 2**

*Supplemental Source Descriptions of the Games in the Present Review*

| Game Name | Supplemental Source | Method of Identification |
| --- | --- | --- |
| *ACTIVATE* | Bikic et al. (2015) | References |
| *Adaptive ICT* | Trial registration (NCT03363568) | Cited in source article |
| *adhD* | Hashemian & Gotsis (2013) | Provided by corresponding author |
| *AixTent* | Sturm et al., 2003 | Database search |
| *ATHYNOS* | Avila-Pesantez et al. (2018) | Database search |
| *Bio Trace+* | Unavailable | N/A |
| *Boogies Academy/ Cuibrain* | Garmen et al. (2019) | References |
| *Braingame Brian* | Prins et al. (2013) | References |
| *Cogmed WMT* | N/A | N/A |
| *CogoLand* | Lim et al. (2023) | Database search |
| *CPAT* | Kolodny et al. (2017) | Database search |
| *EndeavorRx* | N/A | N/A |
| *Eye-contact Tracking Game* | Unavailable | N/A |
| *Focus Pocus* | N/A | N/A |
| *HappyNeuron Pro* | Menascu et al. (2021) [Sup 1] | Database search |
| *IBBS* | Smith et al. (2019) | Database search |
| *N-back Training* | Katz et al. (2014) | References |
| *Plan-It Commander* | N/A | N/A |
| *RECOGNeyes* | Waitt (2021) | Database search |
| *Shape Up* | Hwang et al. (2019) | Database search |
| *SmartMind* | Moradi et al. (2024) | Database search |
| *Supermecha* | Unavailable | N/A |

*Note.* “Supplemental sources” include product descriptions, study protocols, book chapters, dissertations, and secondary studies not otherwise captured by our systematic review. We did not search for a supplemental description of *CogMed*, *EndeavorRx*, *Focus Pocus* or *Plan-It Commander* because three or more studies met our criteria for primary source materials and appeared sufficient. No supplemental sources of information could be found for *Bio Trace+* or *Supermecha*.

Adaptive ICT = Adaptive Inhibitiory Control Training; adhD = Adventurous Dreaming Highflying Dragon; CPAT = Computerized Progressive Attentional Training; IBBS = Integrated Brain, Body, & Social Intervention

**Supplemental Source References**

Avila-Pesantez, D., Vaca-Cardenas, L., Rivera, L. A., Zuniga, L., & Avila, L. M. (2018, April). Athynos: Helping children with dyspraxia through an augmented reality serious game. In *2018 International Conference on eDemocracy & eGovernment (ICEDEG)* (pp. 286-290). IEEE.

Bikic, A., Leckman, J. F., Lindschou, J., Christensen, T. Ø., & Dalsgaard, S. (2015). Cognitive computer training in children with attention deficit hyperactivity disorder (ADHD) versus no intervention: Study protocol for a randomized controlled trial. *Trials*, *16*(1), 1-13. <https://doi.org/10.1186/s13063-015-0975-8>

Garmen, P., Rodriguez, C., Garcia-Redondo, P., & San-Pedro-Veledo, J. C. (2019). Multiple intelligences and video games: Assessment and intervention with TOI software. *Comunicar: Media Education Research Journal*, *27*(58), 95-104.

Hashemian, Y., & Gotsis, M. (2013, November). Adventurous dreaming highflying dragon: A full body game for children with attention deficit hyperactivity disorder (ADHD). In *Proceedings of the 4th Conference on Wireless Health* (pp. 1-2). <http://dx.doi.org/10.1145/2534088.2534101>

Hwang, J., Lee, I.M., Fernandez, A.M., Hillman, C. H., & Lu, A.S. (2019). Exploring energy expenditure and body movement of exergaming in children of different weight status. *Pediatric Exercise Science*, *31*(4), 438-447.

Katz, B., Jaeggi, S., Buschkuehl, M., Stegman, A., & Shah, P. (2014). Differential effect of motivational features on training improvements in school-based cognitive training. *Frontiers in* *Human Neuroscience*, *8*, 242. https://doi.org/10.3389/fnhum.2014.00242

Kolodny, T., Ashkenazi, Y., Farhi, M., & Shalev, L. (2017). Computerized progressive attention training (CPAT) vs. active control in adults with ADHD. *Journal of Cognitive Enhancement*, *1*, 526-538.

Lim, C.G., Soh, C.P., Lim, S.S.Y., Fung, D.S.S., Guan, C., & Lee, T.S. (2023). Home-based brain–computer interface attention training program for attention deficit hyperactivity disorder: A feasibility trial. *Child and Adolescent Psychiatry and Mental Health*, *17*(1), 15.

Menascu, S., Aloni, R., Dolev, M., Magalashvili, D., Gutman, K., Dreyer-Alster, S., Tarpin-Bernard, F., Achiron, R., Harari, G., & Achiron, A. (2021). Targeted cognitive game training enhances cognitive performance in multiple sclerosis patients treated with interferon beta 1-a. *Journal of NeuroEngineering and Rehabilitation*, *18*, 1-8.

Moradi, N., Rajabi, S., & Mansouri Nejad, A. (2024). The effect of neurofeedback training combined with computer cognitive games on the time perception, attention, and working memory in children with ADHD. *Applied Neuropsychology: Child*, *13*(1), 24-36.

Prins, P. J., Brink, E. T., Dovis, S., Ponsioen, A., Geurts, H. M., De Vries, M., & Van Der Oord, S. (2013). “Braingame Brian”: Toward an executive function training program with game elements for children with ADHD and cognitive control problems. *Games for Health: Research, Development, and Clinical Applications*, *2*(1), 44-49.

Smith, S. D., Crowley, M. J., Ferrey, A., Ramsey, K., Wexler, B. E., Leckman, J. F., & Sukhodolsky, D. G. (2019). Effects of Integrated Brain, Body, and Social (IBBS) intervention on ERP measures of attentional control in children with ADHD. *Psychiatry Research*, *278*, 248-257.

Sturm, W., Fimm, B., Cantagallo, A., Cremel, N., & North, P. (2004). Computerized training of speciﬁc attention deﬁcits in stroke and traumatic brain-injured patients: A multicentric efﬁcacy study. In *Applied Neuropsychology of Attention* (pp. 379-394). Psychology Press.

Waitt, A. E. (2022). *Autonomic and central nervous system correlates of cognitive control training for attentional disorders* (Doctoral dissertation, University of Nottingham).
