# Supplementary material for: A systematic review and content analysis of serious video games for children with ADHD
Source: Front Psychiatry. 2025 Oct 6;16:1605744. doi: 10.3389/fpsyt.2025.1605744 (PMC12536224; doi:10.3389/fpsyt.2025.1605744)
Supplement: Supplementary file 3 [file Table3.docx]

**Supplement 3: Codebook for Videogame Articles**

Cognitive Training Codes:

|  | **Code** | **Task Name** | **Operational Definition of Original Laboratory Task** | **Example Game Modifications** | **Closely related concepts/terms** |
| --- | --- | --- | --- | --- | --- |
| 1 | **CPT** | **Continuous Performance Task** | The examinee must either quickly respond to or ignore target stimuli (e.g., letters) that are presented briefly, one at a time, in an unpredictable sequence over an extended period. Stimuli meant to evoke a response (“Go” stimuli) and stimuli meant to be ignored (oddball stimuli) are categorically similar (e.g., both are letters), requiring the examinee to quickly **discriminate** between the two (e.g., *T* versus *X*).  *Special Note. We will code CPT when the task includes frequent oddball stimuli (≈80%) and rare Go stimuli (≈20%). Or in lieu of proportional information, we will code CPT if this definition is met, and the authors’ stated goal is to train attention (sustained attention or distractibility).*  (<https://youtu.be/KL88UmPa2V0?t=152>) | Stimuli changed from letters (or possibly sounds) to game-themed elements.  Stimulus presentations might *partially* overlap (i.e., one stimulus enters the screen as another exits).  The duration of the CPT could vary from brief (e.g., 30s) to protracted (e.g., 14 mins). | Sustained attention; Selective attention; Distractibility |
| 2 | **GNG** | **Go/No-Go Task** | The examinee must either quickly respond to or ignore target stimuli (e.g., letters) that are presented briefly, one at a time, in an unpredictable sequence over an extended period. Stimuli meant to evoke a response (“Go” stimuli) and stimuli meant to be ignored (oddball stimuli) are categorically similar (e.g., both are letters), requiring the examinee to quickly **discriminate** between the two (e.g., *T* versus *X*).  *Special Note. We will code GNG when the task includes rare oddball stimuli (≈20%) and frequent Go stimuli (≈80%). Or in lieu of proportional information, we will code GNG if this definition is met, and the authors’ stated goal is to train inhibitory control.*  (<https://youtu.be/HIJ2tA-R9uc>) | Stimuli changed from letters (or possibly sounds) to game-themed elements.  Stimulus presentations might *partially* overlap (i.e., one stimulus enters the screen as another exits).  The duration of the GNG could vary from brief (e.g., 30s) to protracted (e.g., 14 mins). | Response inhibition |
| 3 | **SS** | **Stop (Signal) Task** | A visual stimulus is presented, and the examinee must quickly respond according to a characteristic of that stimulus (e.g., direction [left or right]), but must **suspend** all responding during a specified period (e.g., immediately after a tone is presented).  (<https://youtu.be/KAfHh8-Q41s>) | Stimuli are game themed and the characteristic the player is attending to could vary.  Stop signal is game themed and might vary over time. | Response inhibition |
| 4 | **Cnc** | **Cancellation Task** | Examinee must **find** and eliminate (e.g., cross out) target stimuli from among distractor stimuli presented simultaneously.  *Special Note. We will code Cnc when stimuli are presented simultaneously (full overlap) rather than sequentially, like in other tasks (e.g., Go/No Go).*  (<https://youtu.be/HRrIs9YMomQ?t=499>) | Stimuli are game themed, and the act of “elimination” might involve blasting or otherwise removing objects from the game. | Visual perception; Vigilance (aka Bourdon–Wiersma test) |
| 5 | **FT** | **Flanker Task** | Several visual stimuli (e.g., arrows) are presented simultaneously, and the examinee must **identify the direction** (e.g., pointing left or right) of a single central or target stimulus, while ignoring those that flank it on either side.  (<https://youtu.be/GEgR-JtcMVc>) | Stimuli are game themed.  Distractor stimuli might appear anywhere around the target stimuli (above/below), and directionality could include any orientation (up/down) | Response inhibition  (aka Eriksen Flanker task) |
| 6 | **Strp** | **Stroop Task** | The examinee is presented with color names (e.g., “red,” “blue”) written in congruous or incongruous font color and is required to **identify** the font color while ignoring the color name (i.e., inhibiting the interference caused by color-word inconsistency). Emphasis is on speed and accuracy.  (<https://youtu.be/reUic5tyVwo?t=45>) | Stimuli are game themed.  The game might present an alternative set of conflicting attributes other than color/word; or require the player to choose congruent or incongruent options from among many presented onscreen. | Selective attention; Automaticity |
| 7 | **DS** | **Digit Span** | The examinee must **recall** a simple numeric sequence in order, or in reverse order, after presentation.  (<https://youtu.be/M-ymgcaXn-4>) | Rather than numerals, the player might be presented with alternatives, like images of dice or dominoes.  Recollection might be demonstrated by typing or sorting onscreen “manipulatives.” | Memory span; Short-term memory; Working memory |
| 8 | **RS** | **Running Span** | A series of numbers/letters are presented, one after another, and the examinee must **recall** the final *n* items.  (<https://youtu.be/Gn2SypOsR4g>) | Rather than alphanumeric stimuli, the player might have to recall a series of game-themed stimuli that are like letters/numbers.  Recollection might be demonstrated by typing or sorting “manipulatives.”  The recalled sequence might be from the beginning or middle of the series. |  |
| 9 | **CS** | **Complex Span** | A series of numbers/letters are presented, one after another, interspersed with secondary decisions to be solved simultaneously (e.g., math operations, sequencing); the examinee must then **recall** the series.  (<https://youtu.be/HRrIs9YMomQ?t=388>) | Rather than alphanumeric stimuli, the player might have to recall a series of game-themed stimuli that are like letters/numbers.  Recollection might be demonstrated by typing or sorting “manipulatives.” | Working memory (aka Letter-number sequencing) |
| 10 | **nB** | ***n*-back Task** | A series of numbers are presented, one after another, and the examinee must **recall** the number *n* items ago.  (<https://youtu.be/PWHq95-hkkA?t=312>) | Rather than numerals, the player might be presented with alternatives, like dice or dominoes. | Working memory; Working memory capacity |
| 11 | **ST** | **Simon Task** | The examinee is taught a relationship between a stimulus (e.g., circle, square) and a simple directional response (e.g., left/right), and then must **quickly respond** accordingly to a series of stimuli, regardless of their location on the computer screen. For example, the left stimulus might appear on the left side of the screen (congruent), or it might appear on the right side of the screen (incongruent).  (<https://youtube.com/shorts/HHHqvlJpXNI?feature=share>) | Stimuli are game themed.  The screen positive might vary (top/bottom).  The player might be rewarded for consistent response times across congruent/incongruent conditions. | Cognitive interference |
| 12 | **DLT** | **Deary-Liewald Task** | The examinee must **quickly respond** to stimuli appearing randomly, one at a time, in one of four fixed boxes/locations. The examinee responds using four response buttons corresponding to the four boxes/locations. Emphasis is placed on speed and accuracy.  (<https://youtu.be/X8Wo8UjmvgY?t=92>) | Stimuli are game themed.  The number of boxes or locations might vary, perhaps beginning with one and increasing over time.  Response device might vary (e.g., keyboard, joystick). | Reaction time; Hick’s Law (aka Choice reaction time; Choice task) |
| 13 | **Cd** | **Coding** | The examinee must **match** a simple symbol to a digit, across multiple trials, based on a key of ≈10 one-to-one relationships provided in advance; emphasis is placed on speed and accuracy.  (<https://youtu.be/HRrIs9YMomQ?t=346>) | Symbols are game themed.  Instead of numbers, players might match the symbol to letters or other familiar stimuli.  The key might use slightly more or less than 10 relationships. | Attention; Motor speed (aka Digit symbol substitution test; Digit symbol coding) |
| 14 | **CT** | **Corsi (Block-Tapping) Task** | The examinee is presented with a pattern of visually presented stimuli (possibly in a matrix) and must **mimic** the spatial pattern, forwards or backwards, after it is completed/removed. In the classic task, the number of stimuli in the pattern increases across successive trials.  (<https://youtu.be/2oKqoZonBkE>) | Stimuli are game themed.  The pattern varies from 2 to 6 stimuli (or even more) | Spatial span; Visuospatial working memory; Finger windows; Visual memory task |
| 15 | **CF** | **Complex Figure** | The examinee must **copy/recreate** a complex visual figure (e.g., multiple lines, squares, circles) while observing a model, or from memory. In the classic task, examinees draw the figure using paper and pencil.  (<https://youtu.be/cRbUA-sY8FM?t=31>) | Stimuli are game themed.  Rather than drawing the figure, a game might require players to move objects into place to assemble the figure. | Visual memory (aka Rey-Osterrieth Complex Figure) |
| 16 | **TT** | **Trail-making Test** | The examinee must **find** circles scattered on a page and sequence them, perhaps by drawing lines/connectors, based on an attribute assigned to each circle (e.g., letters, numbers, letters-and-numbers).  (<https://youtu.be/gUxIbfvKnNk?t=91>) | Stimuli (e.g., circles) and their attributes (e.g., numbers) are game themed.  Rather than lines or connectors, a game might require players to travel, or plan a trip, from point to point. | Visual search; Alternating attention; Sequencing |
| 17 | **WCS** | **Wisconsin Card Sort** | The examinee must **match** cards depicting varying numbers of geometric shapes of different colors, based on either (a) color correspondence, (b) shape correspondence, or (c) number of shapes correspondence. The rule governing the match is unannounced and can occasionally change without notice; the examinee must deduce the rule and adjust to rule changes based on feedback (i.e., correct/incorrect).  (<https://youtu.be/KBVcFEVdKKw?t=13>) | Stimuli are game themed and may not resemble “cards.”  The game might use object characteristics other than shapes, colors, or numbers for the basis of matching.  Feedback might be provided in creative ways (e.g., sounds, images). | Executive functioning; Set shifting; Abstract reasoning |
| 18 | **SC** | **Stockings of Cambridge** | The examinee must **copy/recreate** a modelled pattern of visual stimuli (i.e., colored balls in pockets) using scrambled manipulatives immediately below. Some manipulatives cannot be moved until other manipulatives are taken out of the way, requiring *planning*. Emphasis is placed on copying the model in as few moves as possible (efficiency). In some iterations, examinees are simply asked to predict the number of moves required to recreate the model.  (<https://youtu.be/wpi0l9mE2co?t=25>) | Stimuli are game themed.  The model might appear anywhere onscreen in relationship to the manipulatives.  Players might have to reconstruct the pattern from memory. | Executive functioning; Reasoning and planning abilities (aka Tower of London, Tower of Hanoi) |
| 19 | **OW** | **Controlled Oral Word Association Task** | The examinee must **list** as many words as quickly as possible that belong to a given category (e.g., words beginning with a specific letter).  (<https://youtu.be/8r7t8Qplje8>) | Word categories are game themed.  Rather than saying the words aloud, players might type in the words, identify them from a given list, or by responding to a set of images. | Verbal fluency (aka Category fluency test) |

Neurofeedback Codes:

|  | **Code** | **Task Name** | **Operational Definition of Original Laboratory Task** | **Example Game Modifications** | **Closely related concepts/terms** |
| --- | --- | --- | --- | --- | --- |
| 20 | **NF** | **Neuro-feedback** | The participant controls elements of the game via electroencephalogram (EEG) device for the purpose of increasing beta wave activity, decreasing theta wave activity, etc. | Game themed elements.  Does not need to explicitly target beta/theta waves. | Brain-computer interface |

Behavioral Training Codes:

|  | **Code** | **Task Name** | **Operational Definition of Original Laboratory Task** | **Example Game Modifications** | **Closely related concepts/terms** |
| --- | --- | --- | --- | --- | --- |
| 21 | **BT** | **Behavioral Training** | The game attempts to teach specific skills (e.g., organization, planning, note-taking) intended to address the academic or social impairments of ADHD. | Game themed elements. | Coping skills; Training interventions; Academic enablers; Daily life skills |
