# Supplementary material for: A systematic review and content analysis of serious video games for children with ADHD
Source: Front Psychiatry. 2025 Oct 6;16:1605744. doi: 10.3389/fpsyt.2025.1605744 (PMC12536224; doi:10.3389/fpsyt.2025.1605744)
Supplement: Supplementary file 4 [file Table4.docx]

**Supplement 4***Databases Represented in the Initial Search for Systematic Reviews (Stage 1)*

| Records Found (Overlapping) | Database |
| --- | --- |
| 53 | Scopus |
| 31 | Pubmed |
| 30 | IngentaConnect Databases |
| 30 | Ingenta |
| 25 | Social Sciences Citation Index |
| 25 | DOAJ Directory of Open Access Journals |
| 25 | Science Citation Index Expanded (Web of Science) |
| 23 | ProQuest Central |
| 23 | ROAD: Directory of Open Access Scholarly Resources |
| 20 | PubMed Central |
| 15 | Health & Medical Collection |
| 15 | MEDLINE Current (from 1996) |
| 15 | Health Reference Center Academic |
| 14 | ScienceDirect |
| 10 | Gale Health and Wellness |
| 9 | Ebook Central Perpetual and DDA |
| 9 | eBooks on EBSCOhost |
| 8 | Nursing & Allied Health Database |
| 8 | Research Library |
| 8 | Journals@Ovid Ovid Full Text |
| 8 | SpringerLink |
| 8 | EBSCO_MEDLINE Complete |
| 7 | Psychology Database |
| 7 | ClinicalKey |
| 6 | SpringerLINK |
| 6 | Biological Science Database |
| 5 | APA PsycInfo^†^ |
| 5 | Open Access: Elsevier Open Access Journals |
| 5 | ScienceDirect Health & Life Sciences College Edition Backfile |
| 5 | Backfile Package—Medicine and Dentistry (Supplement 1) |
| 5 | Backfile Package—Medicine and Dentistry (Supplement 1 & 2) |
| 5 | Backfile Package—Medicine and Dentistry (Legacy) |
| 5 | Springer Books |
| 5 | Backfile Package—Psychology (Legacy) |
| 4 | EBSCOhost CINAHL Complete |
| 4 | Advanced Technologies & Aerospace Collection |
| 4 | EBSCOhost :: CINAHL Plus with Full Text (EBSCO) |
| 4 | Engineering Research Database |
| 4 | Freely Accessible Journals |
| 4 | Backfile Package—Psychiatry and Mental Health [YHS] |
| 4 | ScienceDirect Neuroscience Backfile |
| 4 | Journals@Ovid LWW Journal Definitive Archive Collection |
| 4 | Backfile Package—Neuroscience |
| 3 | Applied Social Sciences Index & Abstracts (ASSIA) |
| 3 | Bacteriology Abstracts (Microbiology B) |
| 3 | Biotechnology Research Abstracts |
| 3 | Journals@Ovid LWW Total Access Collection 2023 with Neurology |
| 3 | Agricultural & Environmental Science Collection |
| 3 | Environmental Sciences and Pollution Management |
| 3 | Taylor & Francis eBooks |
| 3 | Journals@Ovid LWW Journal Definitive Archive Collection 2011 |
| 3 | Consumer Health Database |
| 3 | Social Science Database |
| 3 | Journals@Ovid LWW Legacy Archive |
| 3 | Journals@Ovid LWW Comprehensive Archive Collection 2017-2018 |
| 3 | Single Journals |
| 3 | Taylor & Francis Journals Complete |
| 3 | ULAC Taylor & Francis EBS |
| 2 | Hyper Article en Ligne (HAL) |
| 2 | Ecology Abstracts |
| 2 | ProQuest Environmental Science Journals |
| 2 | Springer Nature – Springer Computer Science (R0) eBooks 2023 |
| 2 | Nursing & Allied Health Collection: Comprehensive |
| 2 | Springer Nature – Springer Computer Science eBooks 2019 |
| 2 | Open Access: Freely Accessible Journals by Multiple Vendors |
| 2 | Backfile Package – Computer Science include Supplement 1 |
| 2 | Computer Science Database |
| 2 | Library Science Database |
| 2 | Science Database |
| 2 | Sociology Database |
| 2 | BioMed Central |
| 2 | Mary Ann Liebert Online |
| 2 | Backfile Package – Computer |
| 2 | Backfile Package – Computer Science (Legacy) |
| 2 | SpringerLINK Archive – Medicine |
| 2 | Taylor & Francis Social Science and Humanities Library |
| 1 | Wiley Online Library |
| 1 | SpringerLINK Lecture Notes in Computer Science |
| 1 | Lecture Notes in Computer Science (LNCS) |
| 1 | Biodiversity Heritage Library (Open Access) |
| 1 | Career & Technical Education Database |
| 1 | Industrial and Applied Microbiology Abstracts (Microbiology A) |
| 1 | Toxicology Abstracts |
| 1 | TOXLINE |
| 1 | ABI/INFORM Collection |
| 1 | ProQuest Aquatic Science Journals |
| 1 | ACM Digital Library Complete |
| 1 | Ebook Central – Academic Complete |
| 1 | IEEE Journals Archive 2005-2009 |
| 1 | IEEE Journals Archive 2000-2004 |
| 1 | arXiv Computer Science |
| 1 | eLibrary |
| 1 | Knovel Library |
| 1 | ClinicalKey for Nursing |
| 1 | Middle East & Africa Database |
| 1 | EBSCOhost Business Source Complete |
| 1 | Education Resource Complete |
| 1 | MasterFILE Premier |

***Note***. Our search phrase specified the diagnosis (“ADHD”), clinical focus (“intervention OR treatment OR training OR therap*”), delivery mechanism (“computer* OR digital* OR technolog*”), format (“game OR gamification”), and study type (“meta-analysis OR systematic review”).

^†^ APA PsycArticles and APA PsycInfo databases were not included in OneSearch, so a separate search was conducted in those databases, returning five records, all attributable to APA PsycInfo.
