# Supplementary material for: A systematic review and content analysis of serious video games for children with ADHD
Source: Front Psychiatry. 2025 Oct 6;16:1605744. doi: 10.3389/fpsyt.2025.1605744 (PMC12536224; doi:10.3389/fpsyt.2025.1605744)
Supplement: Supplementary file 5 [file Table5.docx]

**Supplement 5**

*All Excluded Results and Rationale for Exclusion Decisions during the Initial Search for Systematic Reviews (Stage 1)*

| Authors | DOI/ISSN | Reason for Exclusion | Code | Notes |
| --- | --- | --- | --- | --- |
| Agredo-Delgado et al., 2021 | https://link.springer.com/book/10.1007/978-3-030-66919-5 | Not peer reviewed | 1a | Book of conference proceedings |
| Alves et al., 2022 | http://dx.doi.org/10.1089/cyber.2020.0867 | Not focused on treatment games for youth | 1d | Not focused on games |
| Alves et al., 2022 | http://dx.doi.org/10.1089/cyber.2020.0867 | Not focused on treatment games for youth |  | DUPLICATE |
| Anderson, 2020 | https://ieeexplore.ieee.org/ielx7/6/9150532/09150542.pdf?tag=1 | Not peer reviewed | 1a | IEEE article about EndeavorRx |
| Bashiri et al., 2023 | https://doaj.org/article/dca2d0779f744a3d9ff23f9d7ed0995e | Not in English | Hand | Could not find an English translation |
| Batista et al., 2022 | https://doi.org/10.1145/3554364.3559121 | Not a systematic review | 1b | Examines game design issues for ADHD games (focusing on Taboo!) |
| Bickham, 2021 | https://doi.org/10.1007/s40124-020-00236-3 | Unrelated to topic | Hand | Focused on internet addiction |
| Ceranoglu, 2018 | https://doi.org/10.1016/j.chc.2017.11.009 | Unrelated to topic | Hand | Focused on problematic media use |
| Choi et al., 2022 | https://doi.org/10.3389/fpsyt.2022.986687 | Not specific to ADHD | 1c | Includes a wide range of digital therapeutics |
| Cinquin et al., 2019 | https://hal.science/hal-01954983 | Unrelated to topic | Hand | Focused on online e-learning |
| Colzato et al., 2022 | https://doi.o0rg/10.1016/j.neubiorev.2022.104677 | Unrelated to topic | Hand | Focused on ADHD and OCDE |
| Davis et al., 2018 | https://doi.org/10.1371/journal.pone.0189749 | Not a systematic review | 1b | Single study |
| Dong et al., 2020 | https://doi.org/10.1007/s11920-020-01154-3 | Unrelated to topic | Hand | Focused on ADHD and multi-tiered systems of support |
| Drigas et al., 2022 | https://doi.org/10.3390/su141610170 | Not a systematic review | 1b | A commentary on virtual reality and metacognitive training |
| Dullur et al., 2021 | https://doi.org/10.1016/j.jpsychires.2020.12.026 | Unrelated to topic | Hand | Focused on gaming disorder |
| Dullur et al., 2021 | https://doi.org/10.1016/j.jpsychires.2020.12.026 | Unrelated to topic | Hand | DUPLICATE |
| Evans et al., 2021 | https://doi.org/10.1080/23794925.2020.1859960 | Not a systematic review | 1b | Overview of FDA clearance for EndeavorRx |
| Evren et al., 2019 | https://doi.org/10.1080/24750573.2018.1490095 | Unrelated to topic | Hand | Focused on internet gaming disorder |
| Frasson et al., 2023 | https://link.springer.com/book/10.1007/978-3-031-32883-1 | Not peer reviewed | 1a | eBook |
| Haegele et al., 2020 | https://ebookcentral.proquest.com/lib/eastcarolina/detail.action?docID=6023759 | Unrelated to topic | Hand | eBook about physical education |
| Ikezawa et al., 2023 | https://doi.org/10.3390/pediatric15010010 | Not specific to ADHD | 1c | Includes multiple neurodevelopmental disorders |
| Kilmer et al., 2023 | https://doi.org/10.12688/f1000research.129090.2 | Unrelated to topic | Hand | Single study focused on neurodivergent youth |
| Kilmer et al., 2023 | https://doi.org/10.12688/f1000research.129090.2 | Unrelated to topic | Hand | DUPLICATE |
| Ko et al., 2023 | https://doi.org/10.1556/2006.2023.00059 | Unrelated to topic | Hand | Focused on gaming disorder |
| Lamas et al., 2019 | https://doi.org/10.1007/978-3-030-29381-9 | Not peer reviewed | 1a | eBook |
| LeLong et al., 2021 | https://doi.org/10.1186/s12887-021-02916-5 | Unrelated to topic | Hand | Focused on fine motor training |
| Loh et al., 2022 | https://doi.org/10.1016/j.compbiomed.2022.105525 | Not focused on treatment games for youth | 1d | Focused on ADHD diagnosis (not always with games) |
| Lopez et al., 2020 | http://dx.doi.org/10.1016/j.cogsys.2019.09.013 | Unrelated | Hand | Focused on unmanned aerial vehicles |
| Lopez-Fernandez, 2021 | https://doi.org/10.3390/ijerph17051516 | Unrelated to topic | Hand | About internet gaming disorder |
| Lussier-Desrochers et al., 2023 | https://doi.org/10.1007/s41252-023-00319-4 | Not a systematic review | 1b | Single study |
| Mahardika et al., 2021 | https://doi.org/10.26699/jnk.v8i2.ART.p263-269 | Not in English | Hand | Could not find an English translation |
| Martin-Moratinos et al., 2023 | 10.2196/37742 | Unrelated to topic | Hand | Focused on music in video games |
| Matson, 2023 | https://doi.org/10.1007/978-3-031-19964-6 | Not peer reviewed | 1a | eBook about applied behavior analysis |
| Mayer et al., 2019 | https://doi.org/10.1016/j.cogdev.2018.11.002 | Not specific to ADHD | 1c | Focuses on young adults broadly and executive functioning |
| Mulyati, 2023 | Online ISSN: 2477-698X | Not a systematic review | 1b | Single study of "Tanji Adventure" |
| Munambah et al., 2020 | https://doi.org/10.1155/2020/9582795 | Unrelated to topic | Hand | Focuses on the physical play of children with special health care needs |
| Muzwagi et al., 2021 | https://doi.org/10.1097/PRA.0000000000000582 | Unrelated to topic | Hand | Focuses on ADHD and internet gaming disorders |
| Noda et al., 2019 | https://doi.org/10.1186/s13030-019-0164-1 | Unrelated to topic | Hand | Focused on board games for a wide range of players |
| Oh et al., 2024 | https://doi.org/10.1007/s00787-023-02174-z |  |  | Retained |
| Pandian et al., 2021 | https://doi.org/10.1016/j.psychres.2021.113742 | Unrelated to topic | Hand | Although a review, the authors are focused on comparisons to DHI. |
| Paola et al., 2021 | https://doi.org/10.1016/j.procs.2021.07.071 | Not a systematic review | 1b | Single study of "GlyphReader App" |
| Papanastasiou et al., 2020 | https://doi.org/10.1016/j.heliyon.2020.e04250 | Not specific to ADHD | 1c | Focused on neurodevelopmental disorders broadly |
| Pluhar et al., 2019 | http://doi.org/10.2147/PRBM.S208968 | Unrelated to topic | Hand | Focuses on the misuse of media |
| Rajabi et al., 2020 | https://doi.org/10.1080/21622965.2018.1556101 | Not a systematic review | 1b | Single study of neurofeedback game |
| Reffner, 2018 | ISBN: 9780399580239 | Not peer reviewed | 1a | Book review published in a magazine |
| Ren et al., 2023 | https://doi.org/10.1016/j.ridd.2022.104418 | Not specific to ADHD | 1c | Includes multiple neurodevelopmental disorders |
| Rice, 2023 | https://doi.org/10.4324/9781003222132 | Not peer reviewed | 1a | eBook |
| Rodrigo-Yaguas et al., 2021 | https://doi.org/10.1089/g4h.2021.0073 | Not a systematic review | 1b | Study of single game |
| Ruiz et al., 2019 | https://link.springer.com/content/pdf/10.1007/978-3-030-37386-3 | Not peer reviewed | 1a | eBook of selected conference proceedings |
| Salerno et al., 2022 | https://doi.org/10.3390/children9101528 | Unrelated to topic | Hand | Focused on gaming disorder |
| Shaikh et al., 2022 | https://doi.org/10.1007/s13278-022-00888-7 | Unrelated to topic | Hand | Focused on artificial intelligences in healthcare systems |
| Shepard et al., 2023 | https://doi.org/10.1080/21622965.2022.2106436 | Not specific to ADHD | 1c | Focused on heterogenous adolescent samples |
| Stone, 2019 | https://go.exlibris.link/MjN5Trzy | Not peer reviewed | 1a | eBook |
| Sujar et al., 2022 | http://dx.doi.org/10.2196/33884 | Not a systematic review | 1b | A tutorial guide to developing video games |
| Tan et al., 2023 | https://doi.org/10.1007/978-3-031-35708-4_13 | Unrelated to topic | Hand | Focus is on engineering education games |
| Tsiakas et al., 2020 | https://doi.org/10.3233/TAD-200294 | Not specific to ADHD | 1c | A narrative review of cognitive training |
| Vajawat et al., 2021 | https://doi.org/10.1016/j.psychres.2020.113585 | Not a systematic review | 1b | An overview of a selected number of reviews |
| Vinod & Thomas, 2018 | None available | Not peer reviewed | 1a | Book chapter |
| Wadhera & Kakkar, 2022 | ISBN: 9781003165569 | Not peer reviewed | 1a | eBook |
| Zhang et al., 2023 | https://doi.org/10.3389/fpsyg.2023.1028754 | Unrelated to topic | Hand | Focused on self-regulated learning |

***Note***. Not showing the retained reviews. Two additional duplicates of retained reviews were also removed.
Hand = removed prior to screening based on title (unrelated to topic); 1a = not peer reviewed; 1b = not a systematic review; 1c = Not specific to ADHD; and
1d = Not focused on a treatment game for children or adolescents.
