# Supplementary material for: A systematic review and content analysis of serious video games for children with ADHD
Source: Front Psychiatry. 2025 Oct 6;16:1605744. doi: 10.3389/fpsyt.2025.1605744 (PMC12536224; doi:10.3389/fpsyt.2025.1605744)
Supplement: Supplementary file 6 [file Table6.docx]

**Supplement 6**

*Risk of Bias Results for Randomized Trials as Measured by the Cochrane RoB2 Tools*

| **Study** | | **Game** | **Scale** | **D1** | **D2** | **D3** | **D4** | **D5** | **Overall** |
| --- | --- | --- | --- | --- | --- | --- | --- | --- | --- |
| Bakhshayesh et al. (2011) | | Bio Trace+ | ADHD-RS |  |  |  |  |  |  |
| Benzing & Schmidt (2019) | | Shape UP | CBRS |  |  |  |  |  |  |
| Bigorra et al. (2016) | | Cogmed WMT | CBRS |  |  |  |  |  |  |
| Bikic et al. (2017) | | HappyNeuron Pro | ADHD-RS |  |  |  |  |  |  |
| Bikic et al. (2018) | | ACTIVATE | ADHD-RS |  |  |  |  |  |  |
| Bul et al. (2016)* | | Plan-It Commander | BRIEF |  |  |  |  |  |  |
| Chacko et al. (2014) | | Cogmed WMT | DBD-RS |  |  |  |  |  |  |
| Dovis et al. (2015) | | Braingame Brian | DBD-RS |  |  |  |  |  |  |
| Egeland et al. (2013) | | Cogmed WMT | ADHD-RS |  |  |  |  |  |  |
| García-Redondo et al. (2019) | | Boogies Academy | EDAH |  |  |  |  |  |  |
| Green et al. (2012) | | Cogmed WMT | CBRS |  |  |  |  |  |  |
| Johnstone et al. (2010) | | Focus Pocus | DSM-IV Scale |  |  |  |  |  |  |
| Johnstone et al. (2012) | | Focus Pocus | DSM-IV Scale |  |  |  |  |  |  |
| Johnstone et al. (2017) | | Focus Pocus | ADHD-RS |  |  |  |  |  |  |
| Jones et al. (2020) | | *n*-back | CBRS |  |  |  |  |  |  |
| Klingberg et al. (2005) | | Cogmed | DSM-IV Scale |  |  |  |  |  |  |
| Kollins et al. (2020) | | EndeavorRx | ADHD-RS |  |  |  |  |  |  |
| Lim et al. (2019) | | CogoLand | ADHD-RS |  |  |  |  |  |  |
| Meyer et al. (2020) | | AICT | SNAP |  |  |  |  |  |  |
| Rajabi et al. (2020) | | SmartMind | CBRS |  |  |  |  |  |  |
| Shalev et al. (2007) | | CPAT | ADHD-RS |  |  |  |  |  |  |
| Smith et al. (2020) | | IBBS | SNAP |  |  |  |  |  |  |
| Van der Oord et al. (2012) | | Braingame Brian | DBD-RS |  |  |  |  |  |  |
| van Dongen-Boomsma at al. (2014) | | Cogmed WMT | BRIEF |  |  |  |  |  |  |
|  | Low risk | ***Note.*** D1 = Bias due to randomization process; D2 = Bias due to deviations from intended interventions; D3 = Bias due to missing outcome data; D4 = Bias due to measurement of the outcome; and D5 = Bias due to selection of the reported result.  * A sixth domain (“DS”) is measured on the crossover trials version of the RoB2 tool, and those results in this instance were rated *low risk*.  ADHD-RS = Attention Deficit/Hyperactivity Rating Scale (DuPaul et al., 2016); BRIEF = The Behavior Rating Inventory of Executive Function (Gioia et al., 2000); CBRS = Connors Comprehensive Behavior Rating Scales (Conners, 2008); DBD-RS = Disruptive Behavior Disorders Rating Scale (Fosco et al., 2023); DSM-IV Scale = any bespoke rating scale derived from the Diagnostic and Statistical Manual of Mental Disorders, Fourth Edition (American Psychiatric Association, 1994); EDAH = Evaluation of the Deficit of Attention and Hyperactivity scale (Farré & Narbona, 2001); SNAP = Swanson, Nolan, and Pelham Scale (Swanson, 2003) | | | | | | | |
|  | Some risk |  |  |  |  |  |  |  |  |
|  | High Risk |  |  |  |  |  |  |  |  |

|  |  |
| --- | --- |

*Risk of Bias Results for Nonrandomized Trials as Measured by the Cochrane ROBINS-I Tool*

| **Study** | | **Game** | **Scale** | **D1** | **D2** | **D3** | **D4** | **D5** | **D6** | **D7** | **Overall** |
| --- | --- | --- | --- | --- | --- | --- | --- | --- | --- | --- | --- |
| Kollins et al. (2021) | | EndeavorRx | ADHD-RS |  |  |  |  |  |  |  |  |
| Lim et al. (2012) | | CogoLand | ADHD-RS |  |  |  |  |  |  |  |  |
|  | Low risk | ***Note.*** D1 = Bias due to confounding; D2 = Bias in selection of participants; D3 = Bias in classification of interventions; D4 = Bias due to deviations from intended interventions; and D5 = Bias due to missing data; D6 = Bias in measurement of outcomes; and D7 = Bias due to selection of the reported result.   ADHD-RS = Attention Deficit/Hyperactivity Rating Scale (DuPaul et al., 2016) | | | | | | | | | |
|  | Moderate risk |  |  |  |  |  |  |  |  |  |  |
|  | Serious Risk |  |  |  |  |  |  |  |  |  |  |
|  | Critical Risk |  |  |  |  |  |  |  |  |  |  |
